# Hydrophobic Gating of Ion Permeation in Magnesium Channel CorA

**DOI:** 10.1371/journal.pcbi.1004303

**Published:** 2015-07-16

**Authors:** Chris Neale, Nilmadhab Chakrabarti, Pawel Pomorski, Emil F. Pai, Régis Pomès

**Affiliations:** 1 Molecular Structure and Function, The Hospital for Sick Children, Toronto, Ontario, Canada; 2 Department of Biochemistry, University of Toronto, Toronto, Ontario, Canada; 3 Shared Hierarchical Academic Research Computing Network, Department of Physics and Astronomy, University of Waterloo, Waterloo, Ontario, Canada; 4 Department of Medical Biophysics, University of Toronto, Toronto, Ontario, Canada; 5 Department of Molecular Genetics, University of Toronto, Toronto, Ontario, Canada; 6 Ontario Cancer Institute/Princess Margaret Cancer Centre, Campbell Family Institute for Cancer Research, Toronto, Ontario, Canada; University of Illinois, UNITED STATES

## Abstract

Ion channels catalyze ionic permeation across membranes via water-filled pores. To understand how changes in intracellular magnesium concentration regulate the influx of Mg^2+^ into cells, we examine early events in the relaxation of Mg^2+^ channel CorA toward its open state using massively-repeated molecular dynamics simulations conducted either with or without regulatory ions. The pore of CorA contains a 2-nm-long hydrophobic bottleneck which remained dehydrated in most simulations. However, rapid hydration or “wetting” events concurrent with small-amplitude fluctuations in pore diameter occurred spontaneously and reversibly. In the absence of regulatory ions, wetting transitions are more likely and include a wet state that is significantly more stable and more hydrated. The free energy profile for Mg^2+^ permeation presents a barrier whose magnitude is anticorrelated to pore diameter and the extent of hydrophobic hydration. These findings support an allosteric mechanism whereby wetting of a hydrophobic gate couples changes in intracellular magnesium concentration to the onset of ionic conduction.

## Author Summary

This study shows how rapid wetting/dewetting transitions in the pores of ion channels participate in the control of biological ion permeation. Ion channels catalyze ionic permeation across non-polar membranes via water-filled pores. However, non-polar stretches or hydrophobic bottlenecks are present in the pores of many ion channels. To clarify the relationship between channel regulation, pore hydration, and ion permeation, we examine how the slow relaxation of magnesium channel CorA from its closed state towards its open state modulates wetting of its hydrophobic bottleneck. Results provide a quantitative description of wetting and dewetting probabilities and kinetics and a quantitative relationship between the extent of pore hydration and the energetics of ion permeation, consistent with a mechanism of hydrophobic gating.

## Introduction

Magnesium homeostasis is essential for life. In humans, the misregulation of magnesium is implicated in stroke [1], heart disease [2], and diabetes [3]. Magnesium transport is also crucial for bacteria [4]. The movement of magnesium through cell membranes, like that of other ions, is accomplished by integral membrane proteins that provide selective permeability across the dielectric barrier of the lipid bilayer [5]. In bacteria, magnesium uptake is mediated by the CorA protein [6–9], which can substitute for its functional homologue in yeast mitochondria [10]. Electrophysiological data suggests that TmCorA is a channel, not a transporter [8].

**Fig 1:**
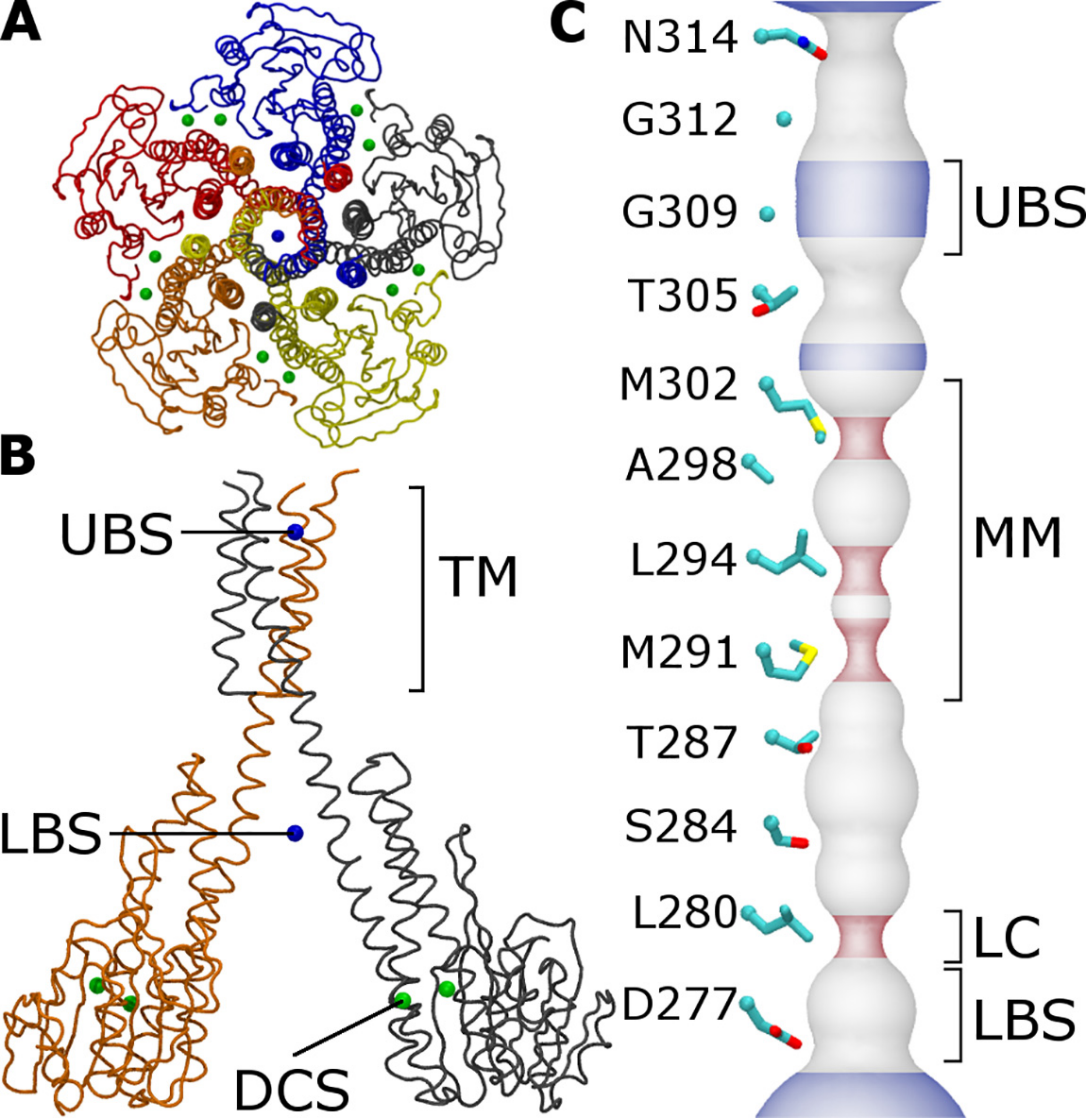
Structural features of the TmCorA protein. (A) Backbone trace of TmCorA viewed along the pore axis from the extracellular side, colored by protomer. Divalent cations in the 2HN2 crystal structure [13] are shown as spheres for (green) regulatory cations and (blue) cations inside the pore. (B) Side view of two protomers. Annotations include cation binding sites in the cytoplasmic domain and the upper and lower regions of the pore (respectively DCS, UBS, and LBS) and the transmembrane region (TM). (C) Surface representation of the pore diameter in the 2HN2 crystal structure [13]. The lower leucine constriction (LC) is indicated. The pore is colored by its local diameter, which is either (blue) larger than that of hexahydrated magnesium (0.68 nm), (red) smaller than that of a single water molecule (0.28 nm), or (grey) intermediate.

Seven crystallographic structures exist for CorA, six of which are from *Thermotoga maritima* (TmCorA) [11–16]. These structures reveal a homopentamer in which 10-nm-long protomeric α-helices (the “stalk” helices) form a transmembrane (TM) pore through which magnesium is presumed to flow. This pore contains two hydrophobic constrictions: the “MM stretch” (MM), a 1.9-nm-long constriction formed by pore-lining residues M291, L294, A298, and M302; and the “lower leucine constriction” (LC), a shorter steric bottleneck formed by the sidechain of L280 (Fig 1). Mutagenesis studies suggest that the MM, but not the LC, is involved in channel gating [17, 18]. Hydrophobic gates are important for the function of many ion channels, including ligand-gated [19–22], voltage-gated [23, 24], phosphorylation-gated [25], and mechanosensitive channels [26, 27].

In all crystal structures of CorA, both hydrophobic constrictions are too narrow to be hydrated, suggesting that the channel is in its closed state. Remarkably, the pore extends beyond the relatively small TM domain into a much larger funnel-shaped domain that protrudes by 6 nm into the cytosol (Fig 1). At the far rim of this funnel, divalent cations are bound between cytosolic protomer interfaces (the divalent cation sensor or DCS; Fig 1). Based on crystallographic structures, it was hypothesized that divalent cation occupancy of the DCS regulates magnesium transport by controlling the pore's diameter or electrostatic profile [11–13]. Recent studies suggest that divalent cation binding to the DCS locks TmCorA in a transport incompetent conformation and that loss of these cations leads to an open conformation of the channel [14, 28], which may be asymmetric [14].

To investigate the allosteric regulation of pore opening, we previously conducted a molecular dynamics (MD) study of TmCorA in a hydrated lipid bilayer, either with or without Mg^2+^ ions in the DCS [29]. The MM remained dehydrated throughout a 110-ns MD simulation in the presence of regulatory ions, but became dilated and hydrated in one of two trajectories generated after these ions were removed [29]. Wetting of the MM involved an iris-like mechanism initiated by the rearrangement of the cytosolic domain interfaces and transmitted to the MM by the long pore-lining stalk helices. These findings suggest a model of allosterically-regulated hydrophobic gating whereby decreasing cytosolic magnesium concentration reduces magnesium occupancy in the DCS, leading to sudden wetting of the pore.

Here, we use massive sampling to examine the statistical significance and the kinetics of this apparent hydrophobic gating process, and to assess whether it results in the open state of the channel. Scaling up computational sampling by two orders of magnitude, we compare hundreds of 35-ns MD simulations in which the DCS are either fully occupied or empty. From a total of 54 microseconds of sampling, we observe many events in which completely connected columns of water condense and evaporate in the MM. We show that this hydrophobic gate is more likely to become hydrated in the absence of regulatory ions and we quantify the kinetics of wetting and dewetting transitions. Finally, we show that the extent of hydration of the MM is correlated to a reduction in the free energy barrier for the permeation of Mg^2+^ ions, supporting the hypothesis that the MM is an allosterically-regulated hydrophobic gate.

## Results

### Hydrophobic Hydration

To determine the kinetics of allosterically-regulated wetting in the MM (the presumed hydrophobic gate), we massively repeated MD simulations of TmCorA in the two limiting states of ionic regulation, namely, with all ten regulatory (DCS) binding sites either occupied or empty. The PDB:2HN2 crystallographic structure of TmCorA [13] was embedded in a hydrated lipid bilayer and seven-hundred 35-ns MD simulations were conducted for each regulatory state. Initially, water filled most of the pore but the MM and the LC were completely dehydrated. Although the MM remained dehydrated in most of the simulations, it was hydrated at least part of the time in 12 and 25% (respectively 83 and 175) of the 700 simulations conducted respectively with and without regulatory ions (Table 1), allowing us to quantify the effect of regulatory ions on pore hydration. MM wetting is depicted as S1 Movie. In wetting transitions, water molecules entered the MM along the pore axis and, occasionally, through transient packing defects between pore-lining helices. The latter defects connected the middle of the pore directly to bulk water at the cytosolic membrane-water interface (S1 Fig).

**Table 1:** Number of simulations in which the MM was wetted for increasingly large percentages of the trajectory.

% of trajectory hydrated (ns in brackets)	*N* simulations meeting criterion		
	Regulatory ions		ratio^a^
	no	yes	
>0 (0)	175	83	2.1
≥0.05 (0.18)	100	31	3.2
≥1 (0.35)	78	25	3.1
≥5 (1.75)	28	6	4.7
≥10 (3.5)	13	4	3.2
≥20 (7)	10	1	10

^a^ *N*(no reg. ions) / *N*(reg. ions)

**Fig 2:**
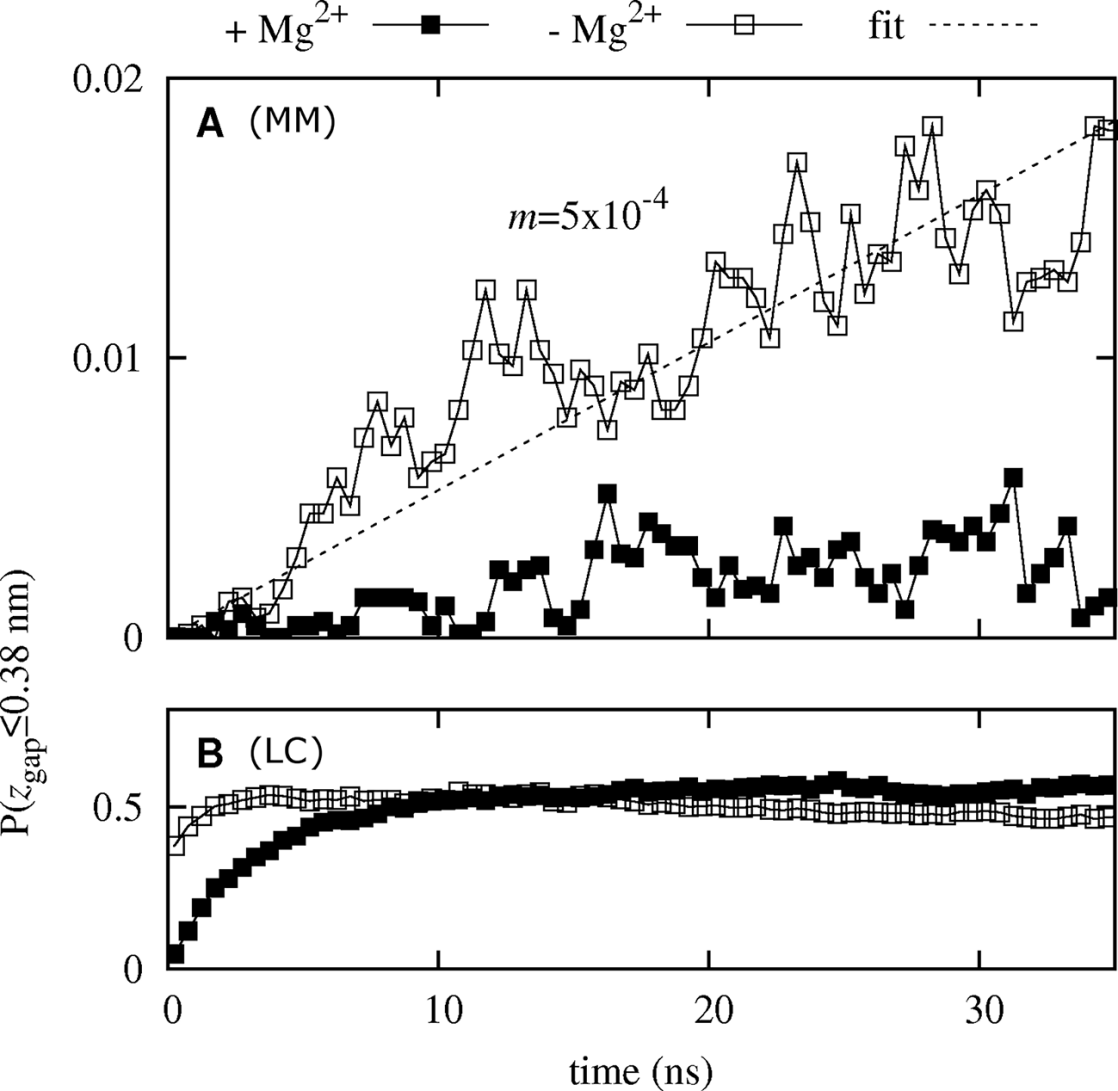
Pore hydration. Fraction of simulations with a completely connected water column, *P*(*z*_gap_≤0.38 nm), in (A) the MM and (B) the LC as a function of simulation time. Results based on all simulations are shown for systems (filled squares) with regulatory ions and (empty squares) without regulatory ions. A linear fit is indicated by a broken line.

To characterize wetting, we computed the maximum distance between consecutive water oxygen atoms along the pore axis, *z*_gap_. Wetting in the MM or LC is defined as end-to-end hydration by a completely connected water column (*z*_gap_ ≤ 0.38 nm). The fraction of simulations in which the MM was wetted, *P*(*z*_gap_ ≤ 0.38 nm), is shown as a function of simulation time in Fig 2A. In the presence of regulatory ions, this fraction appears to stabilize around 0.3% after 15 ns (Fig 2A). In contrast, the MM was wetted five times more often in the absence of regulatory ions, and the wetting probability increased from 0 to 2% through the entire length of the simulations (Fig 2A). Conversely, hydration of the LC increased from 0 to 50% independently of ionic occupancy of the DCS, although equilibration occurred more rapidly in the absence of regulatory ions (Fig 2B).

### Extent of Pore Hydration

To quantify the extent of hydration of the hydrophobic constrictions, we used two metrics, the length of the longest dehydrated stretch, *z*_gap_, and the number of water molecules, *N*_wat_. As expected, these two metrics are strongly anti-correlated (S2A and S2E Fig). The normalized distributions of *z*_gap_ (S2B and S2F Fig) and *N*_wat_ (S2C and S2G Fig) in the MM were similar for the two sets of simulations. Hydration defects were most common near M291, at the cytosolic end of the MM (S2D and S2H Fig). These findings are consistent with our previous simulations [29] and with the hypothesis of Lunin *et al*. that M291 and L294 form the main hydrophobic gate [11]. Finally, S3 Fig shows that hydration of the MM and LC pore constrictions are not correlated with each other.

**Fig 3:**
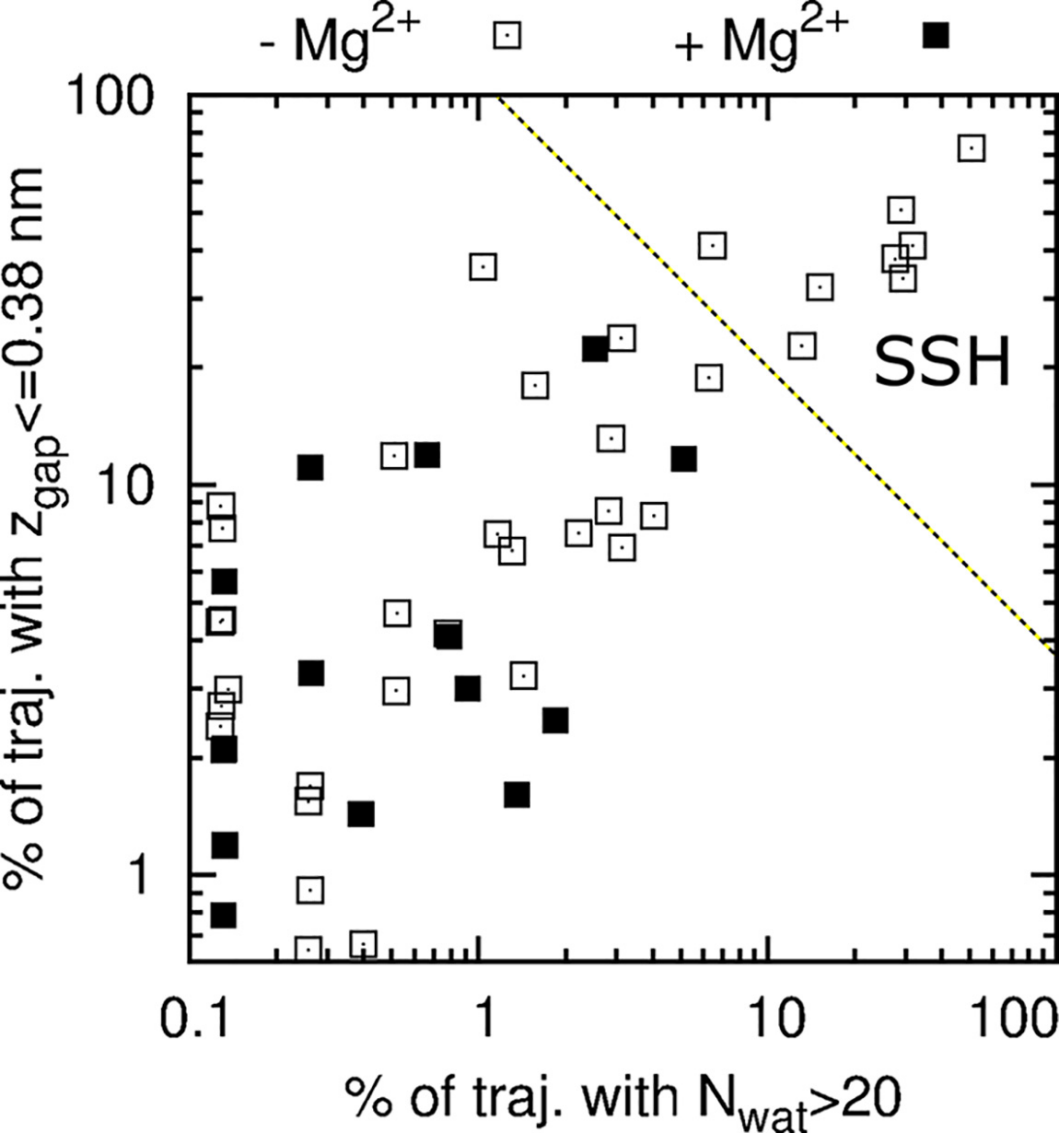
Stability vs. extent of hydration. The percentage of each simulation in which *N*_wat_>20 is plotted against the percentage of that simulation in which *z*_gap_≤0.38 nm. Each point represents a simulation in which regulatory ions are present (filled squares) or absent (open squares). Note that many of the 1,400 simulations are not visible because their MM remained dry. The dividing line between transiently and stably superhydrated (SSH) simulations was selected by eye.

In both regulatory states, many of the wetting events were transient, half of them lasting less than 200 ps. For each simulation, Fig 3 shows the fraction of time that the MM was wetted as a function of the fraction of time that the MM contained >20 water molecules. This analysis leads us to identify eight simulations in which the MM was both stably and highly hydrated, all of which occurred in the absence of regulatory ions. These simulations are henceforth referred to as “stably superhydrated” (SSH).

**Fig 4:**
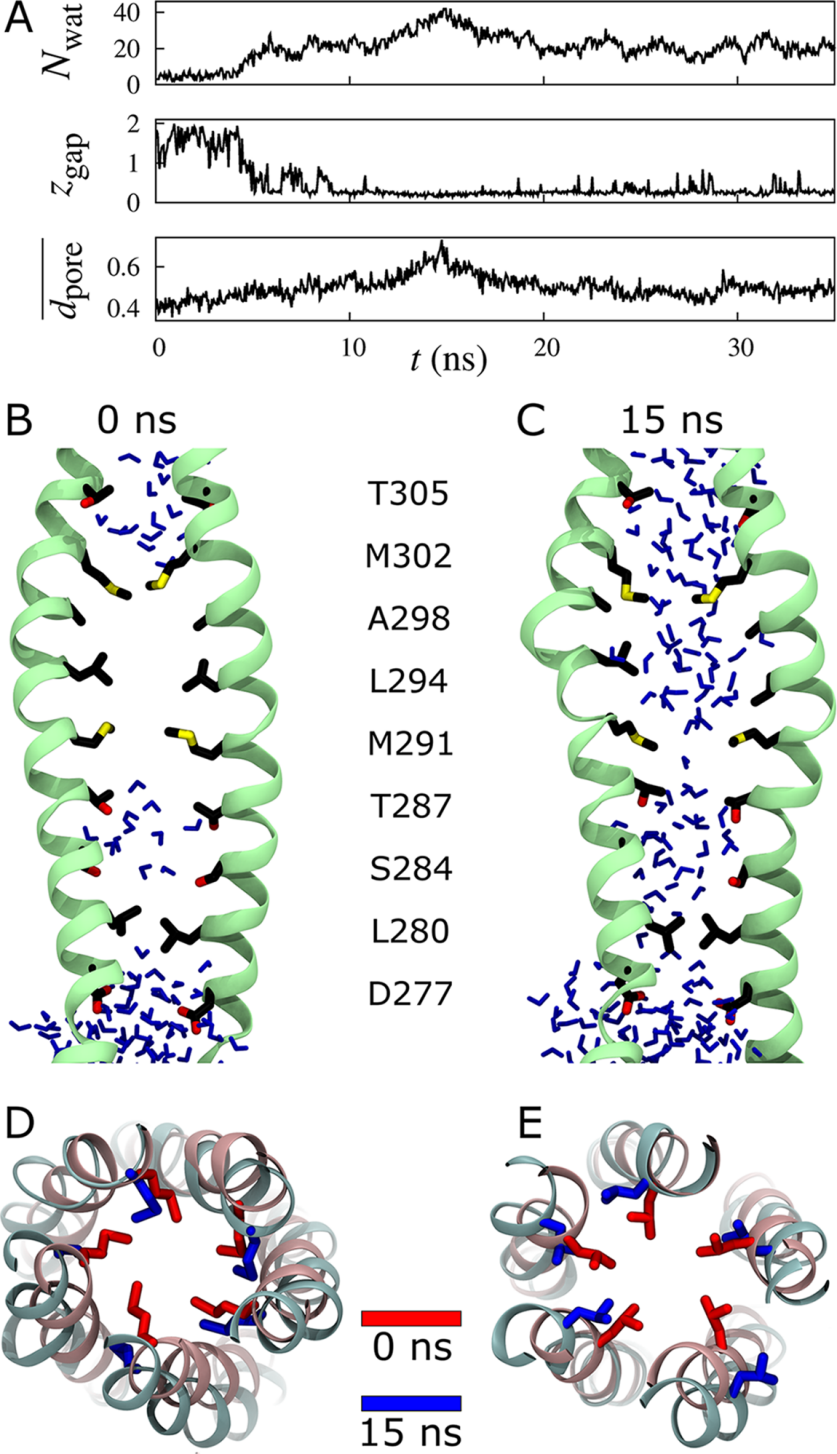
Wetting and pore dilation in the most hydrated SSH simulation. (A) Number of water molecules, *N*_wat_, length of the largest dehydration, *z*_gap_, and average pore diameter, $\bar{d_{\text{pore}}}$, in the MM as functions of simulation time *t* (*z*_gap_ and $\bar{d_{\text{pore}}}$ are in nm). Similar plots for all eight SSH simulations and representative simulations with and without regulatory ions are shown in S4 and S5 and S6 Figs, respectively. (B, C) Side view of two TmCorA protomers highlighting pore-lining sidechains and lumenal water molecules at (B) 0 and (C) 15 ns. (D, E) Extracellular view along the pore axis showing sidechains of (D) M302 and (E) L294 at (red) 0 and (blue) 15 ns.

In the most hydrated SSH simulation, the initially dehydrated MM abruptly and durably filled with ≥20 water molecules, at one point surging to twice this amount (Fig 4A). This hydration was concurrent with an increase in mean MM pore diameter from 0.4 to 0.74 nm (Fig 4A–4C) and involved asymmetric reorientation of pore-lining sidechains away from the lumen, toward neighboring protomers (Fig 4D and 4E).

**Fig 5:**
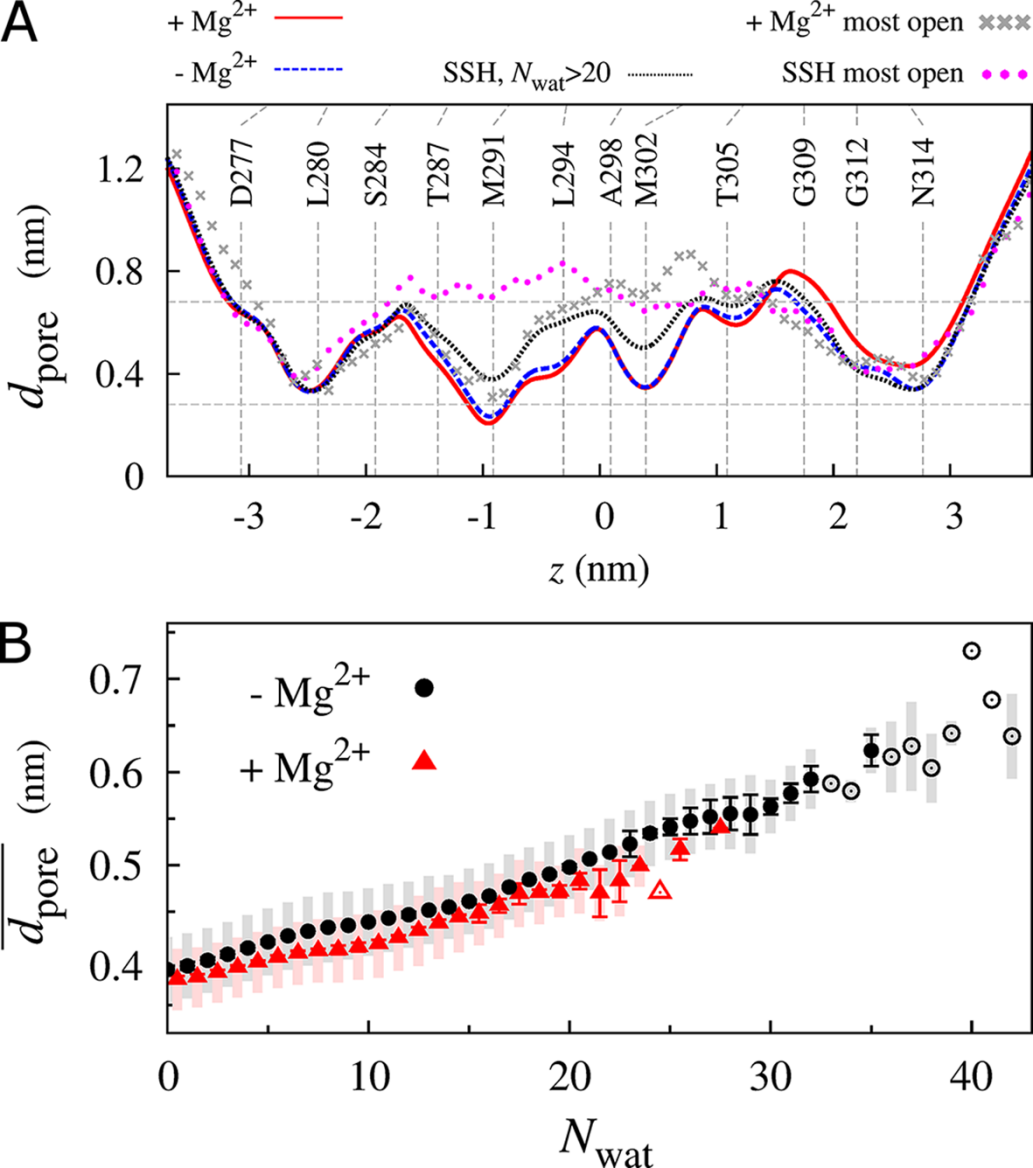
Swelling and hydration of the pore. (A) Pore diameter, *d*_pore_, along the pore axis, *z*. Average values from all simulations (solid red line) with and (broken blue line) without regulatory ions; (dotted black line) conformations with *N*_wat_>20 in all eight SSH simulations; and single conformation with the largest values of mean pore diameter in the MM, $\bar{d_{\text{pore}}}$, from simulations (grey x symbols) with and (magenta circles) without regulatory ions. Vertical lines indicate average positions of terminal heavy atoms of pore-lining sidechains, connecting above residue labels to lines that extend to average C_α_ atom positions. Horizontal lines indicate the diameters of hexahydrated Mg^2+^ and water. The value *z* = 0 corresponds to the center of mass of MM backbone atoms. (B) $\bar{d_{\text{pore}}}$ as a function of *N*_wat_ for simulations (red triangles) with and (black circles) without regulatory ions. Shaded regions enclose one standard deviation of sampled values and error bars represent the uncertainty as the standard deviation of the mean after dividing the trajectories into two sets. Empty symbols denote an inability to assess the uncertainty because the corresponding value of *N*_wat_ was only sampled in one simulation block. Data for simulations with regulatory ions are offset by +0.5 *N*_wat_ units for clarity.

To assess whether hydration was coupled to reorganization of the pore, we computed the pore diameter along its long axis. On average, the volume available in the MM did not substantially depend on regulatory ion occupancy in the DCS (Fig 5A). However, SSH simulations displayed an increase in MM diameter concurrent with wetting. The mean pore diameter in the MM occasionally fluctuated above 0.68 nm (the diameter of a hexahydrated Mg^2+^ ion) in the absence of regulatory ions, but never in their presence (Fig 5A). Upon removal of these ions, there was, on average, a tightening of the pore at its extracellular end, near the proposed selectivity filter of the GMN motif [30] (Fig 5A). Regardless of regulatory ion occupancy, the mean pore diameter in the MM was linearly correlated to the extent of hydration (Fig 5B).

It has been suggested that MM wetting could be triggered by the exposure of previously-hidden hydrophilic moieties to the pore lumen following axial rotation of the stalk helices [15, 16]. To test this hypothesis, we analyzed protein-water hydrogen bond interactions in the MM (S1 Text and S7 and S8 Figs). Taken together, our analysis indicates that pore wetting is not due to a decrease in the hydrophobic character of the MM but rather that its likelihood increases with the volume of the hydrophobic stretch. Accordingly, the increase in both wetting probability and hydration number with CorA pore size is consistent with hydrophobic wetting in simple nanoscopic systems [31] and in hydrophobic pore analogs such as carbon nanotubes [32]. The sharp dependence of hydrophobic wetting on dynamic fluctuations in pore diameter makes this process well suited to gating in a biological channel.

**Fig 6:**
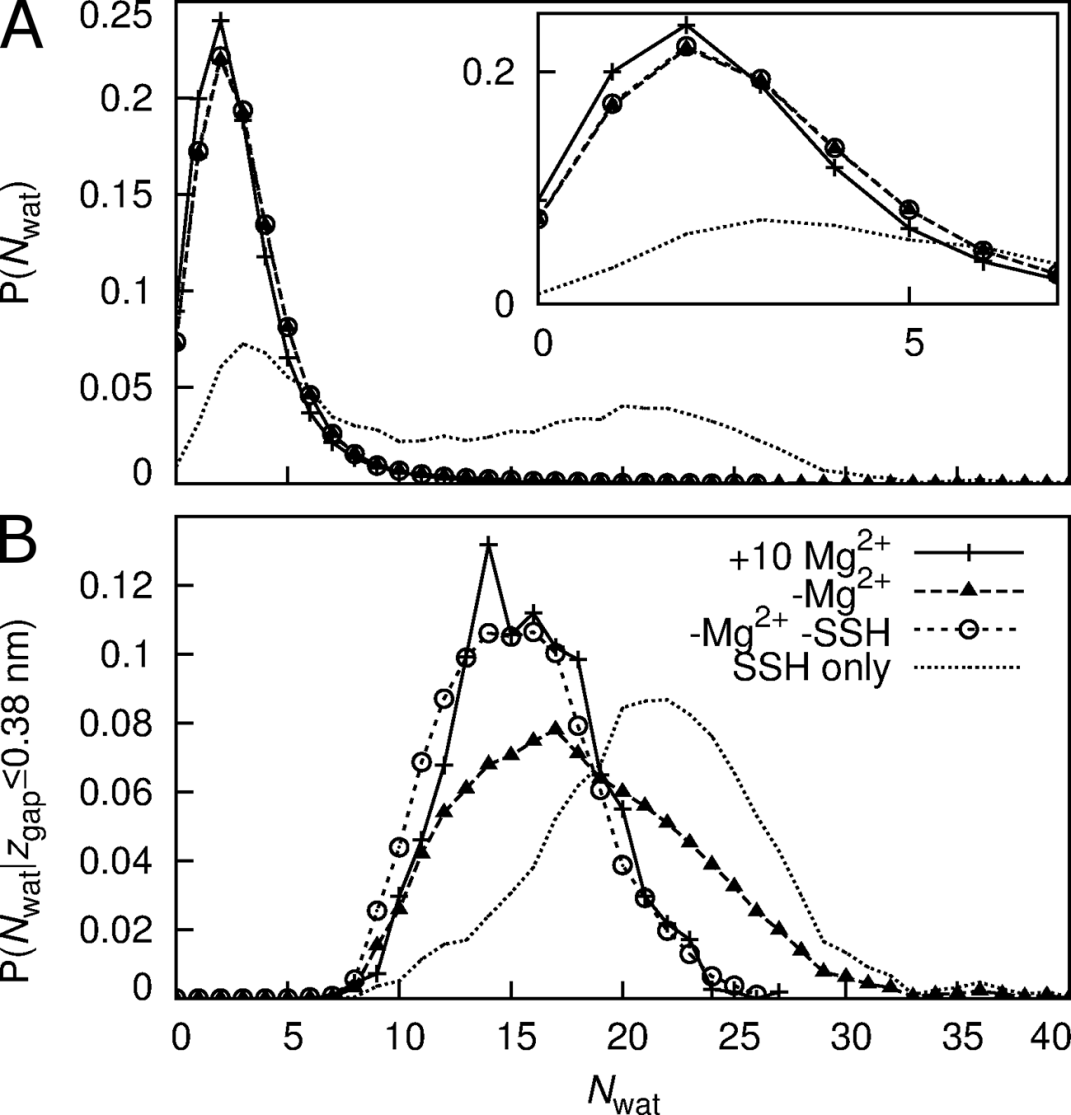
Distribution of *N*_wat_ in the MM. (A) all conformations and (B) only those conformations with a wetted MM; for systems (plus symbols and solid line) with regulatory ions and (filled triangles and broken line) without regulatory ions. Dotted line represents this probability for the entire duration of the 8 simulations that sampled SSH states. Empty circles and dashed line represents this probability for the system without regulatory ions after removing the simulations that sampled SSH states from the analysis.

Distributions of *N*_wat_ are shown in Fig 6A. Despite the presence of wetting transitions, these distributions are unimodal and values of *N*_wat_ greater than 10 are rare. The most likely value of *N*_wat_ in the MM is 2 for simulations both with and without regulatory ions (Fig 6A). The distribution of *N*_wat_ is shifted to slightly larger values in the absence of regulatory ions, a shift that persists after the removal of the SSH simulations from this analysis (Fig 6A). The distribution of *N*_wat_ computed exclusively from the SSH simulations is bimodal, with maxima at *N*_wat_ = 3 and 20 (Fig 6A). Fig 6B shows the distribution of hydration number computed separately for the wetted state. In the SSH simulations, *N*_wat_ is distributed asymmetrically around 22, while the remaining simulations, with and without regulatory ions, sampled values distributed normally around 15 (σ = 3). Thus, in the wet state, the dependence of the extent of hydration on regulatory ions was entirely due to the eight SSH simulations.

### Hydrophobic Wetting Kinetics

**Fig 7:**
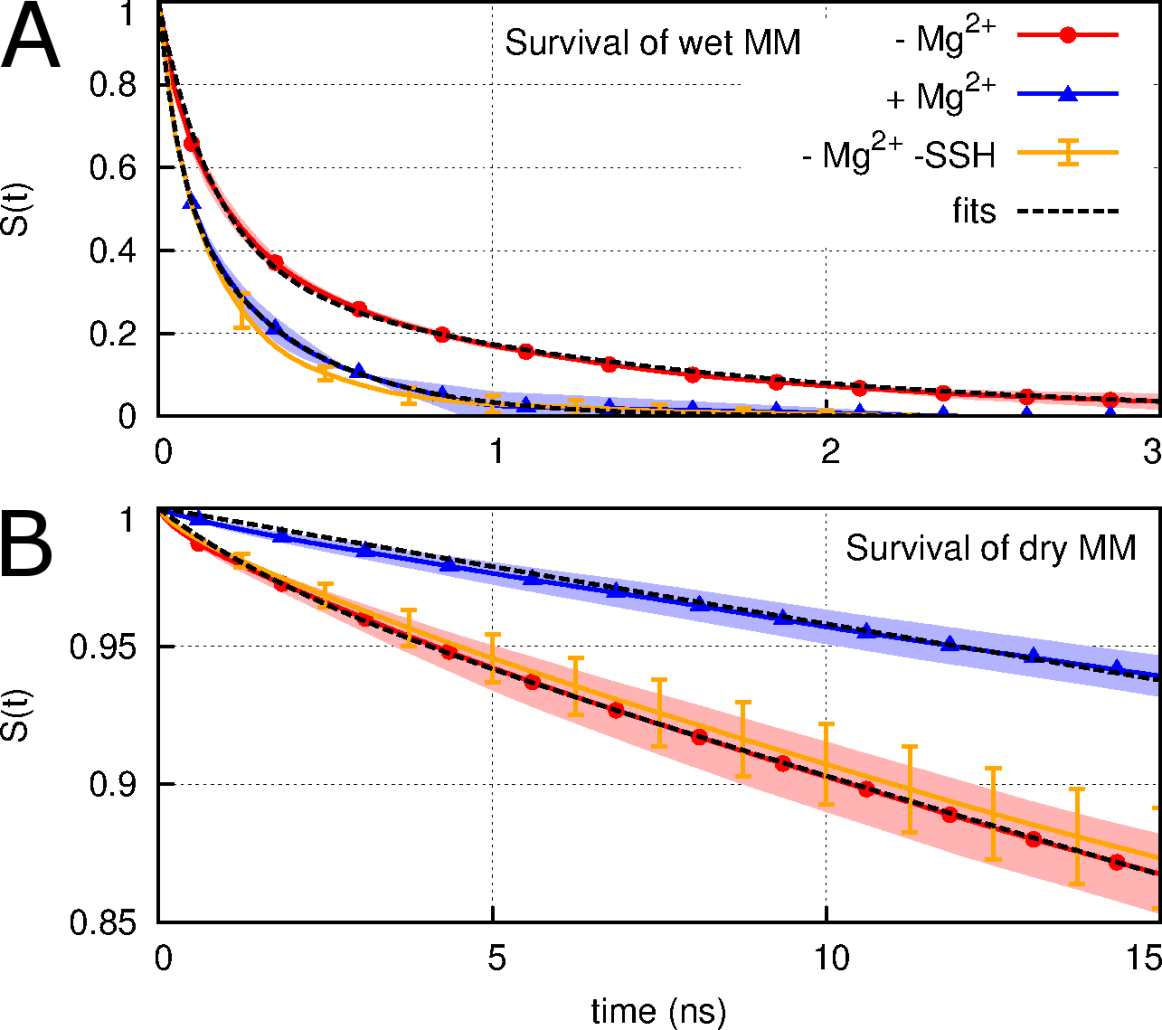
Survival probabilities. (A) completely connected water columns and (B) partial dehydration in the MM; for systems (blue line) with regulatory ions and (red line) without regulatory ions. The orange line was computed for the system without regulatory ions after removing the simulations that sampled SSH states from the analysis. Error estimates are the standard deviations obtained after dividing each set of 700 simulations into two groups.

To characterize the kinetics of wetting and dewetting of the hydrophobic MM, survival probabilities, *S*(*t*), were computed separately for wet and dry states, either with or without regulatory ions (Fig 7). Although wet states are more stable in the absence of regulatory ions, the difference between survival probabilities obtained with and without regulatory ions is abrogated by removing the SSH simulations from this analysis (Fig 7A). Thus, an essential difference between the two sets of simulations is that removal of the regulatory ions led to a hydration state not observed on the 35-ns time-scale in the presence of regulatory ions. The two wetted states differ both in the extent of hydration and in their kinetic properties. Conversely, the dewetted state of the MM was destabilized by the removal of regulatory ions, an effect that persists after removing the SSH simulations from this analysis (Fig 7B).

The survival probabilities of the wetted and dewetted MM states were fit to a double exponential decay function from which we computed the rate of channel wetting to be 8.2×10^6^ ± 7×10^5^ s^-1^ and 4.1×10^6^ ± 3×10^5^ s^-1^ in the absence and presence of regulatory ions, respectively (S1 Table). The estimates of wetting and dewetting rates were used to compute free energy differences between hydrated and dehydrated states (S2 Table), which we used to construct a model of hydrophobic gating in CorA (see Discussion).

### Energetics of Magnesium Permeation

**Fig 8:**
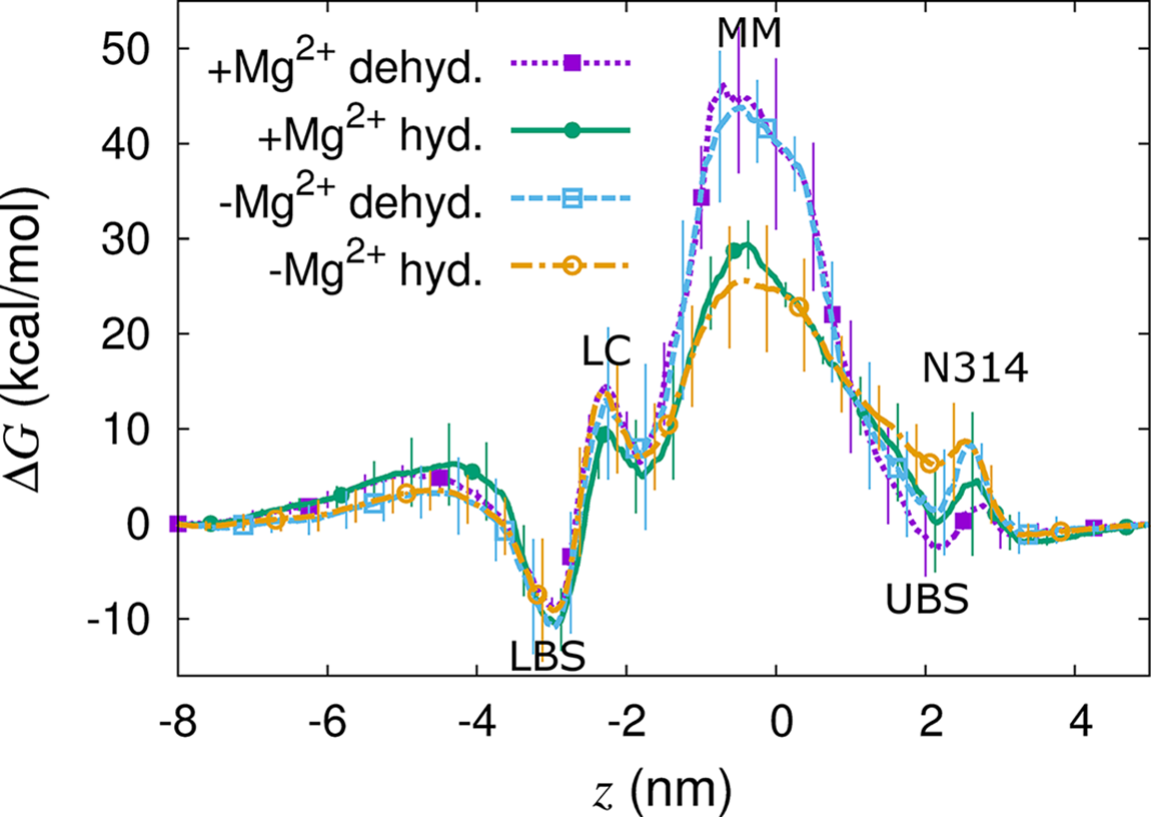
Free energy profiles for a hexahydrated divalent cation through the TmCorA pore. Profiles are shown for conformations in which the MM is (lines with circles) hydrated or (lines with squares) dehydrated, drawn from systems (dotted and solid lines) with regulatory ions and (dash and dash-dot lines) without regulatory ions. The center of mass of backbone atoms in the MM is at *z* = 0 nm. Error estimates are the standard deviation from repeated simulation using different starting conformations.

To assess whether the dilation and hydration of the hydrophobic stretch led to the open state of the channel, we used umbrella sampling (US) [33, 34] to compute the free energy associated with translocation of a divalent cation throughout the pore of TmCorA based on multiple starting conformations from each of four distinct states. These states correspond to hydrated and dehydrated MM with and without regulatory ions. Strictly speaking, our US simulations were conducted out of equilibrium since the channel conformation is slowly relaxing (S9 Fig). As such, these profiles provide estimates of the work required for ion permeation given initial states of protein conformation, pore hydration, and regulatory ion occupancy. Multiple free energy profiles from each state are shown together as ensemble averages in Fig 8. The general features of these profiles are similar: a small barrier at *z* = 2.6 nm, where the sidechain of N314 partially occludes the pore; a barrier of variable magnitude between −1<*z*<1 nm, which corresponds to the MM; a smaller barrier at *z* = −2.3 nm, which corresponds to the LC; and a minimum at *z* = −3 nm, which corresponds to the lower of two pore Mg^2+^ binding sites.

**Fig 9:**
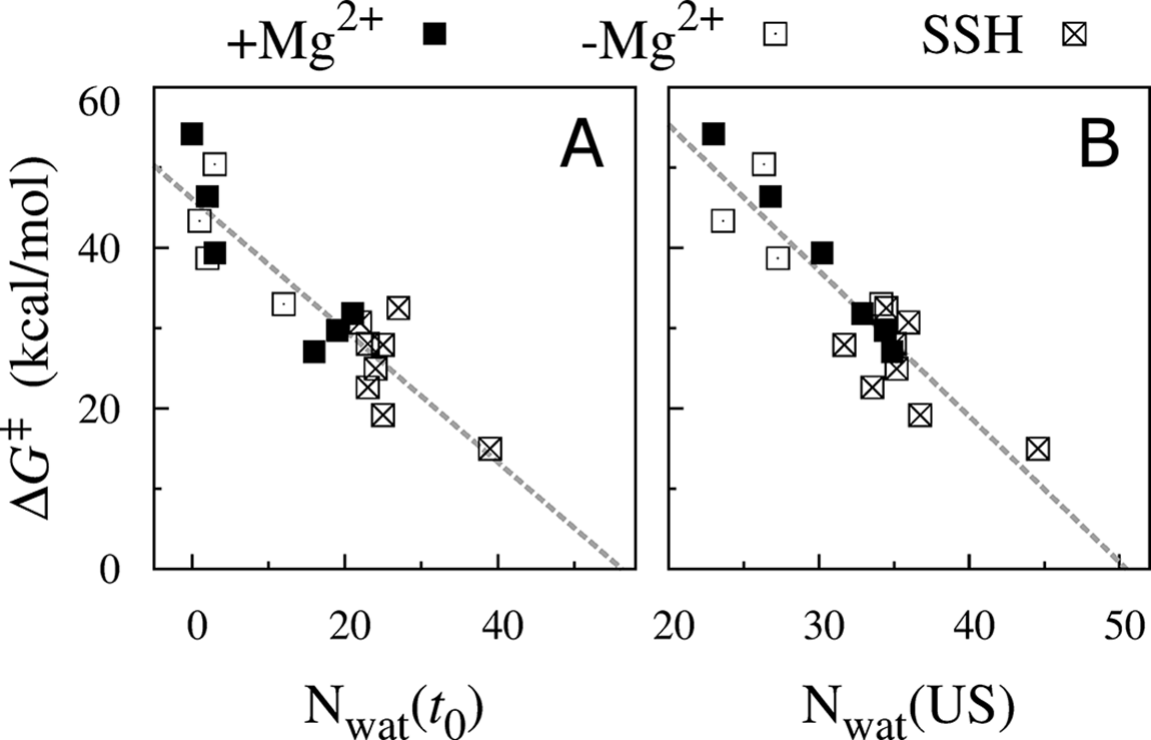
Hydration of the MM is inversely correlated to the work required to move an ion from bulk water to the MM, Δ*G* ^‡^, irrespective of the presence of regulatory ions. Δ*G* ^‡^ is shown as a function of (A) *N*_wat_ from initial US conformations, *N*_wat_(*t*_0_), or (B) the average value of *N*_wat_ from production US while the lumenal Mg^2+^ ion was in the MM, *N*_wat_(US). Data from free energy profiles obtained (filled squares) with and (open squares) without regulatory ions. SSH simulations are identified by an x. Linear fits are indicated by dashed lines. Profiles of *N*_wat_ as functions of lumenal Mg^2+^ position are shown in S10 Fig.

The main difference between the four ensemble-averaged free energy profiles shown in Fig 8 is the height of the barrier in the MM. When the MM is fully dehydrated, it presents a very large barrier of 45±5 kcal/mol irrespective of regulatory binding site occupancy (Fig 8). The size of this barrier reduced to 25±5 kcal/mol when the MM was wetted, again irrespective of bound regulatory ions (Fig 8). Thus, the size of the free energy barrier in the MM correlates with its hydration and not with the presence of regulatory ions. This conclusion is supported and clarified by Fig 9, in which the magnitude of the free energy barrier in the MM, *ΔG* ^‡^, is plotted against values of *N*_wat_ from initial US conformations, *N*_wat_(*t*_0_), or *N*_wat_(US), the average value of *N*_wat_ from US simulations while the lumenal Mg^2+^ ion was in the MM. The magnitude of *ΔG* ^‡^ is anticorrelated with both *N*_wat_(*t*_0_) and *N*_wat_(US) (Fig 9). Linear fits to all data yield *ΔG* ^‡^ = 46–0.82×*N*_wat_(*t*_0_) kcal/mol and *ΔG* ^‡^ = 92–1.82×*N*_wat_(US) kcal/mol, with r^2^ coefficients of 0.80 and 0.83 respectively. By extrapolating these linear relationships, we predict that reducing the magnitude of the barrier to *ΔG* ^‡^ = 0 kcal/mol requires 56 or 50 water molecules based on the two above equations, respectively. A linear fit of the relation between average MM pore diameter, $\bar{d_{\text{pore}}}$, and *N*_wat_ in the absence of regulatory ions (Fig 5B), $\bar{d_{\text{pore}}}$ = 0.37+0.0067×*N*_wat_ nm, suggests that the aforementioned values of *N*_wat_ correspond to average pore diameters of 0.71 to 0.75 nm, slightly larger than the 0.68 nm diameter of hexahydrated Mg^2+^. In turn, this result suggests that the barrier at the MM gate is essentially due to Mg^2+^ desolvation, including effects beyond its first hydration shell. Note that the predicted diameter at which the gating barrier vanishes is significantly smaller than the ~1.2 nm diameter of the putative open state proposed by Dalmas *et al*. [28]. Furthermore, note that the requirement *ΔG* ^‡^ = 0 is excessive as the channel may conduct Mg^2+^ ions with moderate positive values of *ΔG* ^‡^. According to the model we present above, a pore diameter equal to that of hexahydrated Mg^2+^ corresponds to *N*_wat_ = 46 and *ΔG* ^‡^ = 8 kcal/mol.

### Single-Point Mutation Increasing MM Pore-Width

**Fig 10:**
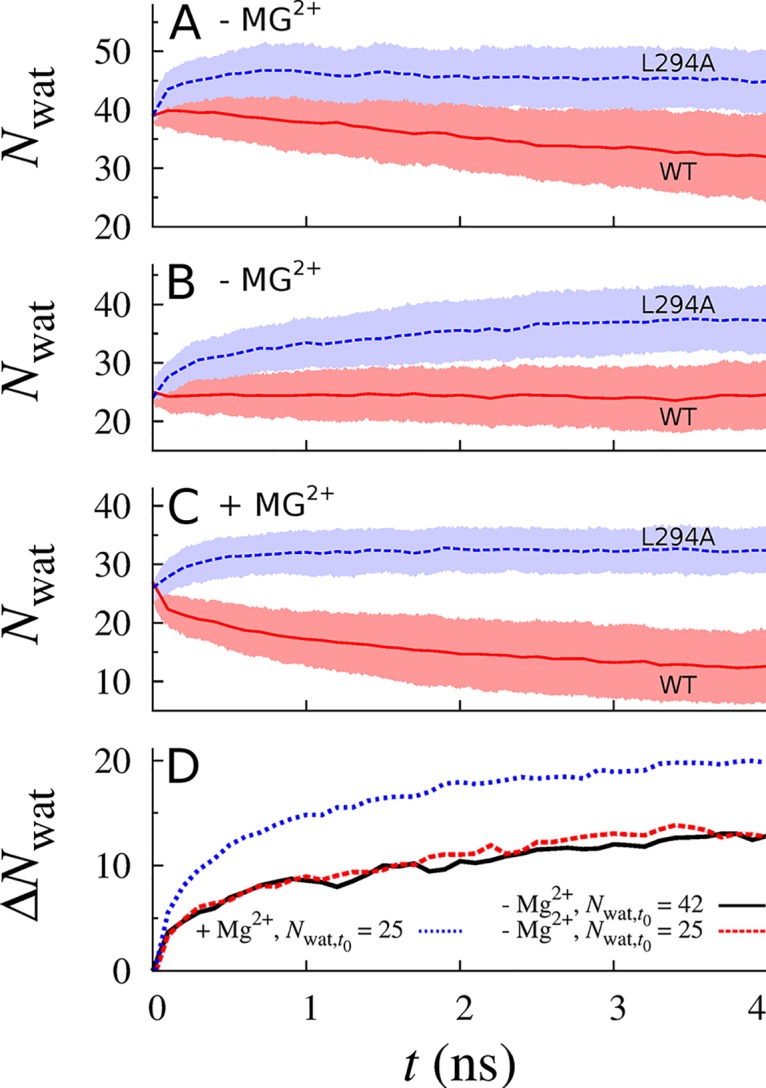
Enhanced MM wetting resulting from pore-widening mutation L294A. (A-C) Ensemble averages of the number of water molecules in the MM, *N*_wat_, as functions of time, *t*, for (solid red) wild type, WT, and (broken blue) L294A simulations (A,B) without and (C) with regulatory ions in the DCS. Shaded regions enclose the standard deviation among 200 repeat simulations. Initial conformations had *N*_wat_ values of (A) 42 or (B, C) 25. (D) Increase in hydration due to the L294A mutation, Δ*N*_wat_ = *N*_wat_(L294A)—*N*_wat_(WT), as a function of *t* (black solid line) without regulatory ions, initial *N*_wat_ = 42; (red broken line) without regulatory ions, initial *N*_wat_ = 25; and (blue dotted line) with regulatory ions, initial *N*_wat_ = 25.

Because pore hydration reduces the free energy barrier to Mg^2+^ flux, we assessed whether removing a (pentameric) bulky hydrophobic sidechain from the MM enhances its hydration. To this end, we conducted two-hundred 4-ns simulations for wild type and L294A TmCorA in three different wetted conformations extracted from our initial set of fourteen-hundred simulations. Consistent with the role of the MM as a hydrophobic gate, the L294A mutation enhances pore hydration both in the presence and in the absence of regulatory ions (Fig 10). In the presence of regulatory ions, L294A simulations initiated with *N*_*wat*_ = 25 stabilized at *N*_*wat*_ = 32 (σ = 4) over the last 3 ns (Fig 10C). Using the linear fit of *N*_*wat*_(*t*_0_) to *ΔG* ^‡^ derived above, we predict that the pore remains closed to Mg^2+^, with *ΔG* ^‡^ = 20 kcal/mol. Therefore, even with the L294A mutation, further conformational rearrangement is required to attain an open state in the presence of regulatory ions. Similarly, wild type simulations initiated with *N*_*wat*_ = 25 or 42 remained stably hydrated with *N*_*wat*_ = 24 (σ = 5) or spontaneously became less hydrated until *N*_*wat*_ = 32 (σ = 7) at 4 ns, respectively (Fig 10A and 10B). Conversely, L294A simulations initiated with *N*_*wat*_ = 25 or 42 approached *N*_*wat*_ values of 37 and 45, respectively (σ = 5; Fig 10A and 10B). Although these extents of hydration predict *ΔG* ^‡^ values between 9 and 16 kcal/mol, the standard deviations of five water molecules among our two-hundred repeat simulations indicates that ~33 simulations initiated with *N*_*wat*_ = 42 continued to sample values of *N*_*wat*_ greater than 50 after 4 ns of simulation, and thus are predicted to present a barrier to Mg^2+^ flux of only 5 kcal/mol. Therefore, in the absence of regulatory ions, the conformational changes that we observe in TmCorA may be sufficient to permit pore opening in the L294A mutant.

### Allosteric Gating Mechanism

We previously proposed that the unbinding of regulatory ions induces changes in the arrangement of protomers in the cytosolic domain of TmCorA which increases the probability of hydration of the MM due to allosteric coupling via the long kinked and tilted helices that wrap around the pore in an iris-like fashion [29]. The present work confirms these conformational changes. Specifically, removing all ten regulatory Mg^2+^ ions results in increased separation between the centers of mass of adjacent Mg^2+^ binding domains (S11A Fig) and decreases the radial tilt angle of the intracellular region of helix 7 (S11B Fig), which together define the width of the funnel-like cytosolic domain. In addition, both the pore radius and the lateral tilt of helix 7 in the MM region increase upon removing regulatory Mg^2+^ ions and further increase in the SSH runs (S11C and S11D Fig).

The first link in this allosteric mechanism is demonstrated by a clear correlation between fluctuations in Mg^2+^ binding domain arrangement and changes in the radial tilt of the pore helices; although this correlation exists regardless of regulatory Mg^2+^ occupancy, these structural fluctuations reach larger amplitudes when the regulatory sites are empty (S12A and S12E and S12I Fig). At the other end of the allosteric transmission, pore diameter and hydration are strongly correlated in the SSH simulations (S12J and S13B Figs), consistent with the above analysis (Fig 5). However, the allosteric mechanism by which we previously proposed radial tilt to cause pore dilation [29] was not strictly maintained throughout our new set of simulations. Specifically, the statistical linkage between average helix tilts and pore diameter is weak when all the SSH runs are considered together (S12L and S12K Fig) and only three out of eight SSH simulations show a significant correlation between average radial and lateral tilts of the pore helices (as defined by a Pearson coefficient magnitude above 0.4; see labels d, e, and h in S13C Fig). Furthermore, our previous description of the iris mechanism [29] involved a positive correlation between average radial and lateral tilts, whereas this correlation is negative in two of the SSH simulations (see labels d and e in S13C Fig), indicating that both increases and decreases in radial tilt of the cytosolic region of helix 7 can lead to pore dilation.

A crystal structure of TmCorA recently obtained in the absence of divalent cations suggests that protomeric asymmetry involving bending of the intracellular domain with respect to the transmembrane region is a precondition for channel opening [14]. Accordingly, the analysis of our simulations shows that asymmetric stalk-helix bending is enhanced by the removal of regulatory ions (S14 Fig). However, there is no correlation between stalk-helix bending and the hydration of the hydrophobic gate (S15 and S16 Figs), suggesting that our 35-ns simulations sample early stages of pore wetting and dilation, and that longer timescales are required for full channel opening.

## Discussion

Like a previous simulation study of protein folding kinetics [35], this study exploited the fact that rare events can be observed from massively-repeated short time trajectories provided that the simulation time exceeds the minimum passage time through the transition state. Our simulations were long enough for wetting and dewetting of the channel to occur, and the number of repeats was large enough to observe hundreds of such transitions, allowing us to derive quantitative estimates of wetting and dewetting kinetics.

Significantly, the MM became hydrated more often when regulatory ions were absent from the simulations (Fig 2). Experimentally, Payandeh *et al*. have shown that a D253F mutation in one of the two pentameric regulatory binding sites enhances bacterial growth in limiting Mg^2+^ conditions [17], presumably because it reduces the ability of these sites to bind Mg^2+^, preventing closure of TmCorA. More recently, Dalmas *et al*. have shown that a D253K mutation abolishes current regulation by the DCS [28]. In addition, our finding that regulatory ions affect hydration of the MM, but not the LC (Figs 2 and 7), suggests that it is the former that is allosterically controlled by regulatory ions in early wetting. However, structural models of the open state generated by Dalmas *et al*. based on spectroscopic data predict an increased pore diameter throughout the entire channel lumen [28], suggesting that longer-timescale pore opening events involve dilation of the LC. In this context, it remains unclear why a pore-widening mutation at the LC (L280A) compromises bacterial growth in limiting Mg^2+^ conditions [17].

The magnitude of the free energy barrier in the MM decreased as it became more hydrated (Fig 9). Consistent with this finding, mutations that shorten the sidechain of MM residue L294 enhance bacterial growth in limiting Mg^2+^ conditions [17, 18] and L294A increases the rate of liposomal uptake of Mg^2+^ by 2.5-fold in a fluorescence-based flux assay [17]. Simulations of the L294A mutant confirm its increased propensity for MM wetting (Fig 10) and suggest that the largest widening of the TmCorA pore observed in our simulations may be sufficient to permit Mg^2+^ flux in the L294A mutant. Our results suggest that these mutations enhance channel activity by increasing the water-accessible volume of the MM region, leading to increased hydration and thereby decreasing the barrier to Mg^2+^ flux.

Hydrophobic gating has been discussed extensively in the literature [36–44]. The transient, reversible wetting of the MM revealed in our simulations (Fig 7) is consistent with the concept that water molecules near hydrophobic surfaces are close to a vapor-to-liquid phase transition [45]. Hydrophobic surfaces alter the phase behavior of water [46], especially when it is tightly confined [31], and wetting equilibria can be dramatically altered by subtle changes in hydrophobicity [32]. Specifically, hydrophobic surfaces enhance water density fluctuations in their vicinity [45, 47], thereby increasing the probability of cavity formation [47]. When such a cavity spans the gap between hydrophobic surfaces, it can nucleate the formation of a vapor phase [31] via capillary drying [48]. Taken together, the above findings suggest that the hydrophobic gate of TmCorA is poised near the edge of a wetting/dewetting transition that can be pushed toward wetting by a single (pentameric) L→A or L→G mutation. Analogous roles have been ascribed to a single (hexameric) V→A mutation in the hydrophobic bottleneck of a calcium release-activated calcium channel [49] and a single I→G mutation that affects the wetting of hydrophobic surfaces during melittin dimerization [45]. In the present study, condensation of water in the MM was correlated to small-amplitude dilations of the pore-lining helices (Figs 4 and 5, S12B, S12F, and S12J Fig). Apolar surfaces only dry when they are sufficiently large [46] and capillary dehydration occurs more rapidly when the enclosing apolar surfaces are closer and longer [31]. At a modal length of 1.9 nm (S2B and S2F Fig) and lumenal diameter on the order of 0.4–0.6 nm (Fig 5), with an average helical spread of 1.54 nm (S12C and S12G Fig), the hydrophobic MM appears to be well constructed to employ capillary dehydration as a gating mechanism.

We observed the following three distinct hydration states in the MM: First, the majority of our simulations remained in a dry state. Second, a wetted state of low hydration, characterized by an average $\bar{N_{\text{Wat}}}$ = 15 (σ = 3), was transiently populated and occurred more often when regulatory ions were absent (Table 1 and Fig 7B and S2 Fig). Although the opening rate of TmCorA is unknown experimentally, the observed rate of wetting in our simulations, 8.2×10^6^ ± 7×10^5^ s^-1^ (S1 Table), is 100–1,000 times faster than the opening rate of any known ion channel, including those involved in fast excitatory neurotransmission such as nicotinic acetylcholine receptors (1×10^4^ to 8×10^4^ s^-1^) [50, 51] and AMPA glutamate receptors (7×10^4^ to 8×10^4^ s^-1^) [52, 53]. Thus, together with the fact that this transient hydrated state is impermeable to Mg^2+^ (Figs 8 and 9), the high rate of wetting suggests that this state is an intermediate. Finally, the third state that we identified, the SSH state (Fig 4 and S4 Fig), differed from transiently-hydrated states in terms of the extent and duration of hydration and in that it was not observed in the presence of regulatory ions (Figs 3, 6 and 7 and S6 Fig).

**Fig 11:**
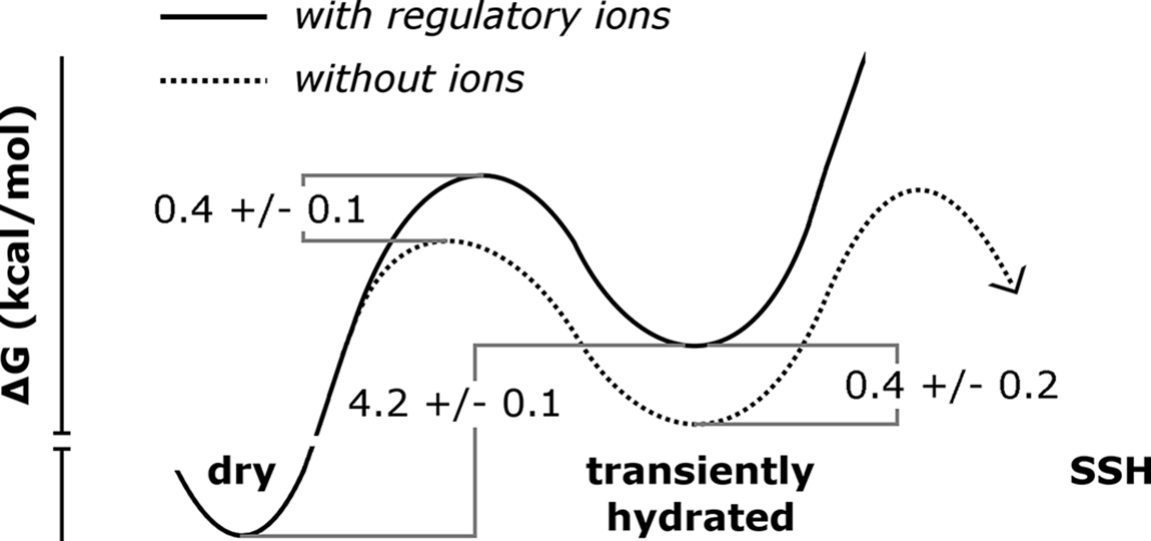
A model of hydrophobic gating in TmCorA.

The overall effect of removing regulatory ions was to increase the extent of hydration in the MM and to increase the stability of wetted states. We propose the following model of wetting and dewetting transitions in the MM, as depicted in Fig 11. In the presence of regulatory ions, the dry state is favored over the transiently hydrated state by 4.2 ± 0.1 kcal/mol (S2 Table). When regulatory ions are removed, the free energy of the transiently-hydrated state, and the barrier to hydration, are reduced by 0.4 ± 0.2 kcal/mol. Regulatory ions thus modulate the rate of wetting but not dewetting transitions respectively to and from the transiently-hydrated state. Furthermore, transitions to the SSH state only occurred in the absence of regulatory ions (Figs 3, 6 and 7, and S6 Fig). This result, together with the linear increase in MM wetting with time (Fig 2A), indicates that equilibrium has not been reached in the absence of regulatory ions and that longer simulations would lead to increasingly divergent fractions of MM wetting based on regulatory ion occupancy.

The steepness of the drop in *ΔG* ^‡^ vs. the extent of hydration, which was observed both with and without regulatory ions (Figs 8 and 9), indicates switch-like behavior. We hypothesize that the removal of regulatory ions stabilizes the SSH state much more dramatically than it stabilizes transiently hydrated states and that the SSH state plays an important role in TmCorA's gating mechanism. Extrapolation of the relationship between *ΔG* ^‡^ and *N*_wat_ suggests that the channel may open to divalent cation flux when more than ~50 water molecules condense in the MM.

Using large-scale sampling, we have characterized the mechanism and kinetics of wetting and dewetting transitions in a 1.9-nm-long hydrophobic constriction of the magnesium channel CorA. Massively-repeated simulations reveal essential differences in the dynamic fluctuations and relaxation of this hydrophobic gate in response to the removal of regulatory ions more than 6 nm away. The analysis of rate constants for wetting and dewetting transitions and of free energy profiles of ionic permittivity indicates that the hydrated states observed in the present study are intermediate states rather than the open state of the channel. These results lead to a model of functional gating of CorA in which regulatory ions control pore hydration, which in turn controls the onset of ionic conduction. A key event underpinning this allosteric mechanism, wetting constitutes a requisite but not sufficient step towards channel opening, as it is the extent of hydration of the pore, not the presence of water *per se*, that determines the barrier to ion permeation. Taken together, these findings demonstrate how capillary wetting transitions help mediate robust, non-linear switching of ionic conduction and underline the relevance of hydrophobic gating to ion channel structure and function.

## Methods

The simulation systems consisted of TmCorA [13], with and without 10 regulatory Mg^2+^ ions in the DCS, in a hydrated 1,2-dimyristoyl-*sn*-glycero-3-phosphatidylcholine (DMPC) bilayer. This system comprised 250,000 atoms. Simulations were performed with GROMACS [54]. The water model was TIP3P [55]. TmCorA was modeled by the OPLS-AA/L parameters [56, 57]. DMPC was modeled by the Berger parameters [58] using the half-ε double-pairlist method [29]. The Mg^2+^ parameters were those of Åqvist [59]. For additional details of system setup and massively repeated simulation, and for a description of the algorithms that we used to quantify hydration, see S2 Text.

To evaluate the free energy profile for the permeation of a divalent cation throughout the pore of TmCorA, we used umbrella sampling (US) MD simulations [33, 34]. In these simulations, we inserted hexahydrated magnesium into the pore, instead of a naked ion, because interactions between magnesium and water molecules in its first hydration shell are very strong [60], suggesting that magnesium is hexahydrated throughout a large part of the conduction pathway. We conducted 261 2-ns simulations in which the axial position of the magnesium ion relative to the center of mass of the hydrophobic gate, *z*, was harmonically restrained to a specified value, $z_{i}^{0}$, for restraining potentials (umbrellas), *i*, distributed every 0.05 nm in the range -8≤$z_{i}^{0}$≤5 nm. These 261 simulations constituted one set of US simulations and were used to generate one evaluation of the free energy profile. Altogether, we conducted 18 sets of US simulations, providing 18 independent evaluations of the free energy profile. In so doing, we computed free energy profiles for four different conformational basins of the TmCorA system, all drawn from our massively repeated sampling. The four basins were: those with the largest and smallest values of *N*_wat_ in the MM, obtained either in the presence or absence of regulatory ions. For each basin, we conducted 3 sets of US simulations, each using a different conformation extracted from a different simulation, except for the large-*N*_wat_ state in the absence of regulatory ions, for which 9 distinct conformations of TmCorA were used to compute 9 free energy profiles. For additional details, see S2 Text.

## Supporting Information

S1 Text Supplementary Results investigate the possibility of pore reorganization involving the exposure of hydrophilic moieties to the pore lumen. (PDF)

S2 Text Supplementary Methods provide detailed descriptions of system setup, massively repeated and umbrella sampling simulations, and algorithms used to quantify hydration. (PDF)

S1 Table Exponential decay fitting parameters and integrals for profiles of survival probabilities, *S*(*t*). Fits are shown separately for (A) the first 350 simulations and (B) the second 350 simulations. (C) Mean lifetime, *τ*. (D) Mean half-life, *t*_1/2_. (E) First-order rate constant, *k*. (n.c. stands for not computed).
(PDF)

S2 Table Free energy values obtained from ratios of the integrals provided in S1 Table. Here, d→tw indicates a transition from the dry state to the transiently wet state.
(PDF)

S1 Fig A snapshot in which a water column transiently connected the interior of the MM to bulk water on the cytoplasmic side of the bilayer. The bilayer is depicted in spheres for (white) carbon, (red) oxygen, (blue) nitrogen, and (brown) phosphorus atoms. Water molecules are shown in cyan. TmCorA is depicted as grey ribbons. Methionine and leucine side chains are shown as yellow and orange sticks, respectively.
(TIF)

S2 FigDistributions of the two hydration metrics *N*_wat_ and *z*_gap_ in simulations (A-D) without regulatory ions and (E-H) with regulatory ions.(A and E) Contour plots correlating *N*_wat_ and *z*_gap_ (Pearson correlation coefficient of -0.8). (B and F) The probability profile of *z*_gap_, in which local minimum at *z*_gap_ = 0.38 nm is indicated by a horizontal line. (C and G) The probability profile of *N*_wat_. In parts B,C,F, and G, both (dashed line) linear- and (solid line) log-scale probabilities are shown, offset for clarity. (*) In part C, probabilities of *N*_wat_ remain non-zero beyond the edge of the plot. (D and H) Inset contour maps of the location of the center of the local dehydration along **z** with respect to the center of mass of the MM, $z_{\text{gap}}^{\text{center}}$, as a function of the length of the dehydrated region. Dotted lines enclose one standard deviation of the positions of the C_α_ atoms of M291 and M302, the residues which bound the MM.(TIF)

S3 Fig Distributions of the hydration metric *z*_gap_ in the MM and LC for systems (A-C) without regulatory ions and (D-F) with regulatory ions. (A and D) Contour plots correlating *z*_gap_ values in the MM and LC. (B and E) The probability profile of *z*_gap_ in the MM. (C and F) The probability profile of *z*_gap_ in the LC. Both (dashed line) linear- and (solid line) log-scale probabilities are shown in parts B,C,E, and F, offset for clarity. A local minimum at *z*_gap_ = 0.38 nm is indicated by horizontal and vertical dotted lines.
(TIF)

S4 Fig Time series of pore hydration in the SSH simulations without Mg^2+^. The number of water molecules in the MM, *N*_wat_, and the length of the largest dehydration, *z*_gap_, are shown. The eight SSH trajectories are ordered A to H by decreasing percent of time spent with *N*_wat_ > 20.
(TIF)

S5 Fig Time series of pore hydration in selected simulations without Mg^2+^. The number of water molecules in the MM, *N*_wat_, and the length of the largest dehydration stretch, *z*_gap_ (nm) are shown. Simulations are ordered by decreasing time spent with *N*_wat_ > 10 and trajectories are shown from the (A) 98^th^, (B) 95^th^, (C) 90^th^, and (D) 50^th^ percentile.
(TIF)

S6 Fig Time series of pore hydration in selected simulations with Mg^2+^. The number of water molecules in the MM, *N*_wat_, and the length of the largest dehydration stretch, *z*_gap_ (nm) are shown for (A-D) the four simulations with the largest time spent with *N*_wat_ > 10 and (E-H) representative trajectories at the 98^th^, 95^th^, 90^th^, and 50^th^ percentile.
(TIF)

S7 Fig Analysis of lumen-facing groups and pore-water interactions in the MM region. (A-D) Dependence of the number of hydrogen bonds between MM residues and lumenal water molecules, *N*_H-bond_, upon the number of water molecules in the MM, *N*_wat_, successively (A, B) without and (C, D) with regulatory magnesium ions. (B, D) Insets show the average value of *N*_H-bond_ as a function of *N*_wat_. (E) Extracellular view of TmCorA showing the snapshot with the largest observed value of *N*_H-bond_ = 7 (not a SSH simulation). Water molecules in the MM that do not engage in water-protein hydrogen bonding are depicted with thinner lines. (F) Orientation of polar backbone groups and side chains of the MM relative to the pore: probability distributions of the (red) backbone O-C-C_COM_ angle; (blue) backbone H-N-N_COM_ angle; (black) lysine N_ζ_-C_α_-C_αCOM_ angle; and (green) threonine Oγ-C_α_-C_αCOM_ angle (in this notation, the subscript _COM_ indicates the center of mass of the five protomeric atoms at a given location). Solid and patterned lines denote simulations without and with regulatory ions, respectively. The appropriate vectors are computed for all MM residues (M291-M302) on all five protomers and combined into a single histogram. (G, H) View of the MM and its hydration from simulations without regulatory magnesium ions in which the carbonyl oxygen atom of F301 accesses the pore, showing snapshots from the (G) least and (H) most hydrated such simulations. MM methionine S_δ_ atoms are shown as yellow spheres and the F301 C = O atoms are shown as cyan and red spheres, respectively. The protein's MM is shown as a cartoon with a colored surface.
(TIF)

S8 Fig Pore reorganization predominantly involves an increase in volume, not in hydrophilic exposure. (A-C) Snapshots depict the (left) least and (right) most hydrated simulation without regulatory ions depicting (A) hydrophilic backbone groups, (B) hydrophilic side chains, and (C) hydrophobic side chains along the MM. (D) Probability that the backbone (red) CO or (blue) NH bond vectors project into the pore (angle ≤ 40° as defined in the caption of S7 Fig) as a function of *N*_wat_.
(TIF)

S9 Fig Convergence of PMFs for ionic conduction. Sampling was initiated using a structure in which the MM was initially (A) wet or (B) dry. Neither of these systems contained regulatory ions. In each case, four PMFs are shown, each computed from 1 ns of sampling per umbrella after an increasing amount of equilibration time per umbrella, *t*_eq_, which was either (solid line) 0, (long-dashed line) 1, (short-dashed line) 9, or (dotted line) 19 ns. (C) The magnitude of the free energy barrier in the MM is shown as a function of *t*_eq_ for the systems in which the MM was initially (“x” symbols and broken line) wet or (open squares and solid line) dry.
(TIF)

S10 Fig Hydration of the MM in US simulations. Each plot depicts a set of US simulations initiated with a different starting structure. Dashed blue horizontal lines indicate the number of water molecules initially in the MM. The average values of *N*_wat_ sampled during US simulations are shown as functions of the position of the lumenal Mg^2+^ ion along the pore axis, *z*, which is centered at the center of mass of MM backbone atoms. Values of *N*_wat_ are averaged over the (red circles) first or (black lines) second ns of simulation/umbrella. Note that the ordinate, *N*_wat_, describes hydration of the MM and not the local environment around the lumenal Mg^2+^ ion.
(TIF)

S11 FigDependence of channel structure on the presence of regulatory Mg^2+^.Data are shown for (dotted black line with filled triangles) all 700 simulations with regulatory ions, (dashed blue line with open circles) the 692 non-SSH simulations without regulatory ions, and (red solid line) the 8 SSH simulations identified in Fig 3 for the system with no regulatory ions. Probability distributions of (A) distances between the centers of mass of adjacent cytoplasmic domains, $d_{MBD}$, (B) radial tilts of the cytoplasmic part of the pore-lining helices, $\theta_{Rad}^{IC}$, (C) lateral tilts of α7 helices on the pore surface in the MM, $\theta_{Lat}^{MM}$, and (D) distances of the axes of α7 helices from the center of the pore in the MM, $d_{center}^{MM}$.(TIF)

S12 FigDependence of structural fluctuations of the channel on the presence of regulatory Mg^2+^.Data are shown for (A-D) all 700 simulations with regulatory ions, (E-H) all 700 simulations without regulatory ions, and (I-L) the 8 stably superhydrated (SSH) simulations identified in Fig 3 for the system with no regulatory ions. Symbols are the same as those in S11 Fig, except that here bars over symbols denote average values from the five protomers. Pairwise correlations are shown for (A,E,I) $\bar{\theta_{Rad}^{IC}}$ *vs* $\bar{d_{MBD}}$, (B,F,J) $\bar{d_{center}^{MM}}$ *vs N*_wat_, (C,G,K) $\bar{\theta_{Rad}^{IC}}$ *vs* $\bar{\theta_{Lat}^{MM}}$, and (D,H,L) $\bar{d_{center}^{MM}}$ *vs* $\bar{\theta_{Lat}^{MM}}$. Regions of dense sampling are colored blue and regions of sparse sampling are colored yellow.(TIF)

S13 Fig Probability distributions of the Pearson correlation coefficients of channel structure and hydration. Pearson coefficients, *ρ*, were computed from individual simulations for the four pairs of metrics considered in S12 Fig. Distributions are shown for (solid line) all 700 simulations without regulatory ions and (broken line) all 700 simulations with regulatory ions. The Pearson correlation coefficient of each of the 8 stably superhydrated simulations is identified in each plot by vertical lines and the letters a-h, which correspond to simulation identifiers in S4 Fig. SSH identifiers are distributed along the ordinate to ease reading, but only contain information in relation to the abscissa.
(TIF)

S14 Fig Stalk helix bending with and without regulatory ions. Probability histograms of the instantaneous (A) minimum, (B) average, and (C) root mean squared deviation (RMSD) of the bending angle between V248, L280, and I310 (all C_α_) in each protomer for simulations conducted (dashed red lines) with and (solid black lines) without regulatory ions. Standard deviations were obtained by dividing each set of 700 simulations into 2 subsets.
(TIF)

S15 Fig Stalk helix bending without regulatory ions. Probability histograms of the instantaneous (A) minimum, (B) average, and (C) root mean squared deviation (RMSD) of the bending angle between V248, L280, and I310 (all C_α_) in each protomer for (solid black lines) all simulations, (dotted red line) not including the SSH simulations, and (dashed blue line) only the SSH simulations. The dotted red line appears almost exactly on top of the solid black line.
(TIF)

S16 Fig Stalk helix bending without regulatory ions as a function of pore hydration. Heat maps evaluate pore hydration via (A-C) *N*_wat_ and (D-F) *z*_gap_ and the instantaneous (A, D) minimum, (B, E) average, and (C, F) root mean squared deviation (RMSD) of the bending angle between V248, L280, and I310 (all C_α_) in each protomer.
(TIF)

S1 Movie Wetting of the hydrophobic gate of TmCorA during a selected 35-ns simulation. Movie depicts TmCorA (grey ribbons) in a lipid bilayer (coloured sticks) and highlights pore hydration by luminal water molecules (yellow spheres). This trajectory represents the SSH simulation in which the MM contained the largest values of *N*_wat_. It was conducted in the absence of regulatory ions.
(AVI)
