# Supplementary material for: Hydrophobic Gating of Ion Permeation in Magnesium Channel CorA
Source: PLoS Comput Biol. 2015 Jul 16;11(7):e1004303. doi: 10.1371/journal.pcbi.1004303 (PMC4504495; doi:10.1371/journal.pcbi.1004303)
Supplement: S1 Text — (PDF) [file pcbi.1004303.s001.pdf]

Supplementary Results

Analysis of MM polarity. It has been proposed that MM wetting involves significant axial rotation of the stalk helices exposing previously-hidden hydrophilic moieties to the pore lumen [1, 2]. To test this hypothesis, we compute the number of hydrogen bonds between the MM and the water molecules that it contains. Although increasing hydration of the MM leads to an increased number of such hydrogen bonds, the average number remains small (≤3) even in the most hydrated states (Fig. S7A-D) and many of these hydrogen bonds involve water molecules moving between helices rather than helical reorganization presenting hydrophilic moieties to the pore lumen. For example, in the single snapshot with the largest number of such hydrogen bonds ($N_{\text{H-bond}}$=7, involving 5 water molecules), four of these hydrogen bonds are formed by two water molecules that partition to the helical interfaces and only three hydrogen bonds involve pore-lining hydrophilic groups (Fig. S7E). The propensity of polar channel groups pointing into the lumen is shown in Fig. S7F. The only polar side chains in the MM, those of lysine and threonine, do not face the lumen. Backbone NH groups are also generally aligned with the helix axis and, as such, are excluded from the pore. Conversely, backbone C=O groups display a small propensity to point into the pore, nearly all of which is due to F301, for which the probability that the O-C-C_{COM} angle is less than 40° is 0.3 ± 0.1 and 0.22 ± 0.02 in simulations without and with regulatory magnesium ions, respectively (in this notation, C_{COM} is the center of mass of the five protomeric carbonyl carbon atoms at a given location). However, note that the F301 C=O moiety lies at the outer edge of the hydrophobic MM (Fig. S7G, H). For all other C=O groups in the MM, the probability that the O-C-C_{COM} angle is less than 40° is ≤0.002.

Further analysis of polar groups in the pore lumen is shown in Fig. S8. Although C=O and N-H groups are sometimes present in the pore lumen (Fig. S8A), as are the side chains of T295 and T299 (Fig. S8B), their presence does not correlate with MM hydration (Figs. S7F and S8A, B, D). Conversely, hydrated states of the pore correspond to a wider spread of hydrophobic side chains in the pore lumen (Fig. S8C). The latter observation is consistent with the correlation between the diameter and the hydration of the MM (see Figs. S11D; S12B,F,J; and S13B).

Taken together, our analysis indicates that pore wetting is not due to a decrease in the hydrophobic character of the MM, but rather, that its likelihood increases with the volume of the hydrophobic stretch.

References

1. Nordin N, Guskov A, Phua T, Sahaf N, Xia Y, Lu S, et al. Exploring the structure and function of Thermotoga maritima CorA reveals the mechanism of gating and ion selectivity in Co2+/Mg2+ transport. Biochem J. 2013;451(3):365-74.
2. Guskov A, Nordin N, Reynaud A, Engman H, Lundbäck A-K, Jong AJO, et al. Structural insights into the mechanisms of Mg2+ uptake, transport, and gating by CorA. P Natl Acad Sci USA. 2012;109(45):18459-64. doi: 10.1073/pnas.1210076109.
