# Supplementary material for: Hydrophobic Gating of Ion Permeation in Magnesium Channel CorA
Source: PLoS Comput Biol. 2015 Jul 16;11(7):e1004303. doi: 10.1371/journal.pcbi.1004303 (PMC4504495; doi:10.1371/journal.pcbi.1004303)
Supplement: S2 Text — (PDF) [file pcbi.1004303.s002.pdf]

Supplementary Methods

Simulation protocol. The simulation systems consisted of CorA from *Thermotoga maritima* (TmCorA) [1] in a hydrated 1,2-dimyristoyl-*sn*-glycero-3-phosphatidylcholine (DMPC) bilayer. Simulations were conducted with version 4.0.5 of the GROMACS simulation package [2]. The water model was TIP3P [3]. TmCorA was modeled by the OPLS-AA/L parameters [4, 5]. DMPC was modeled by the Berger parameters [6] using the half-ε double-pairlist method [7]. The Mg^{2+} parameters were those of Åqvist [8].

Lennard-Jones interactions were evaluated using a group-based cutoff for separation distances less than 1.2 nm and otherwise ignored. Coulomb interactions were calculated using the smooth particle-mesh Ewald method [9, 10] with a real-space cutoff of 1.2 nm and a Fourier grid spacing of 0.14 nm. Simulation in the *NpT* ensemble was achieved by semi-isotropic coupling to Berendsen barostats [11] at 1 bar with coupling constants of 4 ps and temperature coupling using velocity Langevin dynamics [12] at 300 K with a coupling constant of 1 ps. Covalent bonds were constrained with SETTLE [13] and P-LINCS [14] for water and other molecules, respectively. The integration time step was 2 fs. The nonbonded pairlist was updated every 20 fs. Coordinates were saved every 10 ps.

System setup and massively repeated sampling. In our previous study [7], we simulated two of the three crystal structures then available, omitting 2BBJ [15] because it has the lowest resolution. In that study [7], we found that the pore became hydrated in a 110-ns simulation of 2HN2 [1], but not 2IUB [16]. Therefore, the TmCorA protein and associated Mg^{2+} ions were taken from 2HN2 for further study. Because residues are missing at both the N- and C-termini [1], we modeled the crystallographically available first and last residues as neutral -NH_2 and -COOH, respectively. In each of the five protomers, residues 316–325 were missing in the extracellular (EC) loop region connecting the stalk helix (α_7) with the second transmembrane (TM) helix (α_8). We modeled the EC loops with the sequence EYMPELRWKW using the loop prediction program Loopy [17], as in our previous study [7]. The principal axis of the protein was aligned with the normal of a pre-equilibrated DMPC bilayer [18] and adjusted to position the TM domain in the bilayer, as we did previously [7]. A surface representation of the protein

was then constructed using the MSMS algorithm [19] and a TmCorA-shaped hole was made in the bilayer according to the protocol of Faraldo-Gómez *et al.* [20] in 3 consecutive simulations of 20 ps each. During this step, a harmonic restraining potential was applied to the positions of lipid phosphorus atoms along the bilayer normal, with a force constant of 1000 kJ/mol/nm^2. The strength of the hole-making force was, sequentially, 10, 100, and 500 kJ/mol/nm^2. An equilibrated box of liquid water was overlaid on this system and water molecules were removed to resolve steric clashes. The composite system, composed of TmCorA and associated 12 Mg^{2+} ions and crystal water molecules embedded in a solvated DMPC bilayer, was neutralized with 21 Na^+ ions. Position restraints were placed on all protein and Mg^{2+} ions. The system was then subjected to 1000 steps of steepest descent energy minimization and then 800 ps of molecular dynamics (MD) simulation, at which point the position restraints on the modeled protein loop residues 316–325 were released and an additional 1.5 ns of MD was conducted. Finally, all position restraints were removed and two systems were created, one without any Mg^{2+} ions and the other with 10 Mg^{2+} ions in the putative regulatory divalent cation sensor (DCS). Both systems lacked Mg^{2+} ions in the pore. Systems with and without Mg^{2+} ions were neutralized by 4 and 24 additional Na^+ ions, respectively. Harmonic position-restraints were placed on all C_α atoms using a force constant of 1000 kJ/mol/nm^2 and each of these two systems was subjected to 1000 steps of steepest descent energy minimization followed by 500 ps of MD equilibration. Finally, position restraints were removed and each system was simulated for 3 ns, during which conformations were extracted every 30 ps. Each of these 100 extracted conformations for each system was then used to initiate seven 35-ns simulations, each using different initial velocities and random seeds for the Langevin forces. We thus conducted seven-hundred 35-ns simulations either in the absence or presence of regulatory ions. The total simulation time was 49 μs.

Identification of hydrated states. To quantify hydration of the 1.5-nm constriction formed by the pore-lining residues M291, L294, A298, and M302, the MM-stretch or MM, we used a custom algorithm to compute the number of water molecules that satisfied the following conditions: (i) z_A-0.5<z_{wat}<z_B nm, where z_{wat} is the **z** component of the center of mass (COM) of a water oxygen atom, and z_A and z_B are the **z** components of the COM of the N- (residue 291) and C-terminal (residue 302) methionine C_α atoms in the MM, respectively; and (ii) r_{wat}<0.6 nm, where r_{wat} is the radial distance in the **xy** plane from the water oxygen atom to a line that lies at

the center of the pore and connects the COMs of the methionine S_δ atoms that define the ends of the MM. This analysis provides the number of water molecules that reside in the MM, N_{wat}, and the **z** component of the maximum distance between consecutive water molecules, z_{gap}. The calculation of z_{gap} in the steric bottleneck at leucine 280, the lower leucine constriction or LC, was conducted similarly, except that the condition z_C - 0.2 < z_{wat} < z_D + 0.2 nm was used, where z_C and z_D are the **z** components of the COMs of the D277 and S284 C_α atoms, respectively, which flank L280 in the pore lumen. In this case, the central line for the evaluation of r_{wat} connected the C_α atoms of D277 and S284. We did not evaluate N_{wat} in the LC.

The presence of multiple inflection points in the probability distribution of z_{gap} (Figs. S2B, F) but not N_{wat} (Figs. S2C, G) indicates that z_{gap} is a better measure with which to define distinct hydration states: those in which the MM was wetted and those in which the MM contained a hydration defect. Based on the locations of local minima in Figs. S2B and S2F, we set the complete water column threshold at $z_{gap} \leq 0.38$ nm.

The diameter of the pore, d_{pore}, as a function of axial depth was computed with the program HOLE [21]. In this analysis, the presence of very tight constrictions in the MM coupled with transient separation of pore-lining helices (as illustrated in Fig. S1) rarely lead the HOLE program to define the pore as exiting into bulk water near the cytosolic end of the MM. Therefore, for each simulation snapshot, we computed the radial profile with HOLE four times from different initial axial positions along the pore and defined the final radial profile by selecting the diameter value originating closest to the center of the pore's long axis. The average value of d_{pore} in the MM, $\overline{d_{pore}}$, was taken over the same range used in the calculation of N_{wat} and z_{gap}, as outlined above.

Umbrella sampling. To evaluate the free energy profile or potential of mean force (PMF) for the permeation of a divalent cation throughout the CorA pore, we used umbrella sampling (US) MD simulations [22, 23]. To this end, we conducted 261 2-ns simulations in which the axial position of the magnesium ion in relation to the COM of the backbone atoms in the MM (M291, L294, A298, and M302), z, was harmonically restrained to a specified value, z_i^0, for restraining potentials (umbrellas), i, distributed every 0.05 nm in the range $-8 \leq z_i^0 \leq 5$ nm. Thus, 261

simulations constituted one set of US simulations and were used to generate one evaluation of the PMF.

To keep magnesium close to the pore axis at its distal regions, we modified GROMACS version 4.0.5 to allow multiple simultaneous harmonic restraining potentials with different reference groups. Then, in addition to the restraining potential along the Cartesian **z** dimension, we applied a flat-bottomed restraining potential that extended radially from the COM of selected CorA backbone atoms in the Cartesian **xy** plane. When z_i^0 was located on the periplasmic side of the COM of the upper pore Mg^{2+} binding site (UBS; Fig. 1; here defined as residues 304-309), the zero-penalty region of the flat-bottomed restraining potential had a radius of 0.5 nm. Likewise, when z_i^0 was located on the cytoplasmic side of the COM of the lower pore Mg^{2+} binding site (LBS; Fig. 1; here defined as residues 275-280), the zero-penalty region of the flat-bottomed restraining potential had a radius of 1.0 nm. Flat-bottomed restraining potentials were not used for z_i^0 between the UBS and LBS.

All together, we conducted 18 sets of US simulations, providing 18 independent evaluations of the PMF. In so doing, we computed PMFs for four different conformational basins of the TmCorA system, all drawn from our massively repeated sampling. The four basins were: those with the largest, and smallest, values of N_{wat} in the MM, either in the presence or absence of regulatory ions. For each basin, we conducted 3 sets of US simulations, each using a different conformation extracted from a different simulation, except for the large N_{wat} state in the absence of regulatory ions, for which 9 distinct conformations of TmCorA were used to compute 9 PMFs.

Specifically, for each umbrella, hexahydrated magnesium was placed in the radial center of the pore at the specified value of z_i^0 and water molecules initially in the pore were removed if their van der Waals radii overlapped with magnesium hexahydrate. To ensure that magnesium remained hexahydrated during system setup, harmonic restraining potentials were temporarily used to maintain each of the 6 water molecule oxygen atoms in magnesium's first hydration shell near a distance of 0.2 nm from Mg^{2+} using the force constant of the O-H bond in TIP3P water. Each system was then subjected to 5 rounds of steepest descent energy minimization, each of 500 steps, in which the positions of all protein atoms were first frozen, then harmonically restrained, then with harmonic restraints applied only to the positions of backbone atoms, and

then only to C_α atoms, and finally with no restraints on the protein. Next, 20 ps of MD simulation were conducted. The harmonic restraints between the permeating magnesium ion and the water molecules in its first hydration shell were then removed. Post-simulation analysis shows that water molecules in the first solvation shell of the lumenal Mg^{2+} ion were absolutely maintained throughout 9.4 μs of total simulation after harmonic potentials between Mg^{2+} and water were removed. Each umbrella was simulated for 2 ns using an umbrella force constant of 4184 kJ/mol/nm^2. The first 1 ns was discarded as early equilibration based on an analysis of statistical convergence (Fig. S9). The axial position of the permeating magnesium ion, *z*, was stored every 20 fs and the data from each of the 18 sets of US simulations were used, separately, to generate 18 independent PMFs using Alan Grossfield's implementation [24] of the weighted histogram analysis method (WHAM) [25]. To this end, recorded values of the ion insertion depth in the range $-8 \leq z \leq 5$ nm were distributed among 2600 histogram bins and WHAM was run with a tolerance of $1\text{x}10^{-5}$. Because magnesium is in bulk water at both extremes of this PMF, we were able to reduce the accumulation of statistical sampling errors during WHAM by enforcing overlap of the PMF at z=-8 nm and z=5 nm. The PMFs were then shifted to 0 kcal/mol at z=5 nm. The mean free energy, ΔG, and standard deviation of the mean, $\sigma_M = \left[\frac{1}{M-1} \sum_{j=1}^{M} \left(\Delta G_j - \overline{\Delta G} \right)^2 \right]^{\frac{1}{2}}$, for M estimates of ΔG with overall mean $\overline{\Delta G}$, were computed at each histogram bin for each of the 4 conformational basins.

Data analysis. Survival probabilities of the wetted and dewetted MM states were fit to a double exponential decay function of the form $f(t) = a \times \exp(-t/\tau_1) + (1-a) \times \exp(-t/\tau_2)$ for time t. Fitting parameters and the mean lifetimes, τ, of these states, obtained by integrating these functions from 0 to $+\infty$, are provided in Table S1. Also provided in Table S1 are mean half-lives, $t_{1/2}$, computed according to $t_{1/2} = \tau \ln 2$. Although we fit the survival probabilities to a double exponential decay function, the decay of dewetted states were also well represented by a single exponential decay function since a~1 or 0 (Table S1). In these cases, we computed the first-order rate constant, k, of the wetting transition according to $k = \ln(2)/t_{1/2}$.

The ratios of these mean lifetimes for wetting and dewetting transitions provides an estimate of the equilibrium constant, $\langle K_{i,j} \rangle$, and free energy difference, $\Delta G_{j \to i}$, between two

states i and j according to $\langle K_{i,j} \rangle = \frac{1/\tau_i}{1/\tau_j} = \exp\left(-\beta \Delta G_{j \to i}\right)$. Free energy differences between dried and transiently wetted states are listed in Table S2.

Bending of the stalk helices was assessed by using the GROMACS g_angle program to compute the bending angle between V248, L280, and I310 (all C_α) in each protomer.

Hydrogen bonds were computed with the GROMACS g_hbond program using a hydrogen – donor – acceptor angular cutoff of 30° and a hydrogen – donor distance cutoff of 0.35 nm.

The diameter of the TmCorA pore displayed in Fig. 1 was computed using the program HOLE [21]. Molecular visualizations were prepared with VMD [26]. Many figures and all linear fits were constructed with gnuplot.

References

1. Payandeh J, Pai EF. A structural basis for Mg2+ homeostasis and the CorA translocation cycle. EMBO J. 2006;25(16):3762-73.
2. Hess B, Kutzner C, van der Spoel D, Lindahl E. GROMACS 4: algorithms for highly efficient, load-balanced, and scalable molecular simulation. J Chem Theory Comput. 2008;4(3):435-47. doi: 10.1021/ct700301q.
3. Jorgensen WL, Chandrasekhar J, Madura JD, Impey RW, Klein ML. Comparison of simple potential functions for simulating liquid water. J Chem Phys. 1983;79(2):926-35.
4. Jorgensen WL, Maxwell DS, Tirado-Rives J. Development and testing of the OPLS all-atom force field on conformational energetics and properties of organic liquids. J Am Chem Soc. 1996;118(45):11225-36. doi: 10.1021/ja9621760.
5. Kaminski GA, Friesner RA, Tirado-Rives J, Jorgensen WL. Evaluation and reparametrization of the OPLS-AA force field for proteins via comparison with accurate quantum chemical calculations on peptides. J Phys Chem B. 2001;105(28):6474-87. doi: 10.1021/jp003919d.
6. Berger O, Edholm O, Jähnig F. Molecular dynamics simulations of a fluid bilayer of dipalmitoylphosphatidylcholine at full hydration, constant pressure, and constant temperature. Biophys J. 1997;72(5):2002-13.
7. Chakrabarti N, Neale C, Payandeh J, Pai EF, Pomès R. An iris-like mechanism of pore dilation in the CorA magnesium transport system. Biophys J. 2010;98(5):784-92.
8. Åqvist J. Ion-water interaction potentials derived from free energy perturbation simulations. J Phys Chem. 1990;94(21):8021-4. doi: 10.1021/j100384a009.
9. Darden T, York D, Pedersen L. Particle mesh Ewald: An N•log(N) method for Ewald sums in large systems. J Chem Phys. 1993;98(12):10089-92
10. Essmann U, Perera L, Berkowitz ML, Darden T, Lee H, Pedersen LG. A smooth particle mesh Ewald method. J Chem Phys. 1995;103(19):8577-93.
11. Berendsen HJC, Postma JPM, van Gunsteren WF, DiNola A, Haak JR. Molecular dynamics with coupling to an external bath. J Chem Phys. 1984;81(8):3684-90.

12. van Gunsteren WF, Berendsen HJC. A leap-frog algorithm for stochastic dynamics. Mol Sim. 1988;1(3):173-85.
13. Miyamoto S, Kollman PA. Settle: An analytical version of the SHAKE and RATTLE algorithm for rigid water models. J Comput Chem. 1992;13(8):952-62. doi: 10.1002/jcc.540130805.
14. Hess B. P-LINCS: a parallel linear constraint solver for molecular simulation. J Chem Theory Comput. 2008;4(1):116-22. doi: 10.1021/ct700200b.
15. Lunin VV, Dobrovetsky E, Khutoreskaya G, Zhang R, Joachimiak A, Doyle DA, et al. Crystal structure of the CorA Mg2+ transporter. Nature. 2006;440(7085):833-7.
16. Eshaghi S, Niegowski D, Kohl A, Molina DM, Lesley SA, Nordlund P. Crystal structure of a divalent metal ion transporter CorA at 2.9 Angstrom resolution. Science. 2006;313(5785):354-7. doi: 10.1126/science.1127121.
17. Xiang Z, Soto CS, Honig B. Evaluating conformational free energies: the colony energy and its application to the problem of loop prediction. P Natl Acad Sci USA. 2002;99(11):7432-7. doi: 10.1073/pnas.102179699.
18. Gurtovenko AA, Patra M, Karttunen M, Vattulainen I. Cationic DMPC/DMTAP lipid bilayers: molecular dynamics study. Biophys J. 2004;86(6):3461-72.
19. Sanner MF, Olson AJ, Spehner J-C. Reduced surface: an efficient way to compute molecular surfaces. Biopolymers. 1996;38(3):305-20. doi: 10.1002/(sici)1097-0282(199603)38:3<305::aid-bip4>3.0.co;2-y.
20. Faraldo-Gómez JD SG, Sansom MS. Setting up and optimization of membrane protein simulations. Eur Biophys J. 2002;31(3):217-27.
21. Smart O, Goodfellow J, Wallace B. The pore dimensions of gramicidin A. Biophys J. 1993;65(6):2455-60.
22. Torrie GM, Valleau JP. Nonphysical sampling distributions in Monte Carlo free-energy estimation: umbrella sampling. J Comput Phys. 1977;23(2):187-99.
23. Roux B. The calculation of the potential of mean force using computer simulations. Comput Phys Commun. 1995;91(1-3):275-82.
24. Grossfield A. WHAM: the weighted histogram analysis method http://membrane.urmc.rochester.edu/content/wham. accessed September 15, 2014.
25. Kumar S, Rosenberg JM, Bouzida D, Swendsen RH, Kollman PA. The weighted histogram analysis method for free-energy calculations on biomolecules. I. The method. J Comput Chem. 1992;13(8):1011-21. doi: 10.1002/jcc.540130812.
26. Humphrey W, Dalke A, Schulten K. VMD: visual molecular dynamics. J Mol Graphics. 1996;14(1):33-8.
