# Supplementary material for: Hydrophobic Gating of Ion Permeation in Magnesium Channel CorA
Source: PLoS Comput Biol. 2015 Jul 16;11(7):e1004303. doi: 10.1371/journal.pcbi.1004303 (PMC4504495; doi:10.1371/journal.pcbi.1004303)
Supplement: S1 Table — Fits are shown separately for (A) the first 350 simulations and (B) the second 350 simulations. (C) Mean lifetime, τ. (D) Mean half-life, t 1/2. (E) First-order rate constant, k. (n.c. stands for not computed). (PDF) [file pcbi.1004303.s003.pdf]

S1 Table

		$+Mg^{2+}$		$-Mg^{2+}$		$-Mg^{2+}$ -SSH	
		dry	wet	dry	wet	dry	wet
**A**	*a*	0.0072	0.29	0.98	0.95	0.98	0.76
	τ_1 (ns)	2.0	0.62	131	0.23	143	0.13
	τ_2 (ns)	244	0.10	1.3	2.0	1.7	0.60
	τ (ns)	243	0.26	129	0.68	140	0.24
**B**	*a*	0.0030	0.28	0.97	0.56	0.98	0.51
	τ_1 (ns)	0.69	0.036	118	0.13	119	0.29
	τ_2 (ns)	276	0.30	2.6	1.0	2.6	0.068
	τ (ns)	275	0.23	115	0.54	116	0.18
**C**	τ (ns)	259 ± 23	0.24 ± 0.02	122 ± 10	0.6 ± 0.1	128 ± 18	0.21 ± 0.04
**D**	$t_{1/2}$ (ns)	180 ± 16	0.17 ± 0.01	85 ± 6	0.42 ± 0.07	89 ± 12	0.15 ± 0.03
**E**	k (s^{-1})	$3.9x10^6$ ± $3x10^5$	n.c.	$8.2x10^6$ ± $7x10^5$	n.c.	$7.9x10^6$ ± $1x10^6$	n.c.
