# Supplementary material for: Hydrophobic Gating of Ion Permeation in Magnesium Channel CorA
Source: PLoS Comput Biol. 2015 Jul 16;11(7):e1004303. doi: 10.1371/journal.pcbi.1004303 (PMC4504495; doi:10.1371/journal.pcbi.1004303)
Supplement: S2 Table — Here, d→tw indicates a transition from the dry state to the transiently wet state. (PDF) [file pcbi.1004303.s004.pdf]

S2 Table

	Value (kcal/mol)
$\Delta G_{\text{d}\rightarrow\text{tw}}^{\text{-Mg}}$	3.8 ± 0.2
$\Delta G_{\text{d}\rightarrow\text{tw}}^{\text{+Mg}}$	4.2 ± 0.1
$\Delta\Delta G_{\text{d}\rightarrow\text{tw}}^{(\text{+Mg}\rightarrow\text{-Mg})}$	-0.4 ± 0.2
$\Delta\Delta G_{\text{d}\rightarrow\text{tw}}^{\ddagger(\text{+Mg}\rightarrow\text{-Mg})}$	-0.4 ± 0.1
$\Delta\Delta G_{\text{tw}\rightarrow\text{d}}^{\ddagger(\text{+Mg}\rightarrow\text{-Mg})}$	-0.07 ± 0.2
