# Supplementary figures and images for: Hydrophobic Gating of Ion Permeation in Magnesium Channel CorA
Source: PLoS Comput Biol. 2015 Jul 16;11(7):e1004303. doi: 10.1371/journal.pcbi.1004303 (PMC4504495; doi:10.1371/journal.pcbi.1004303)

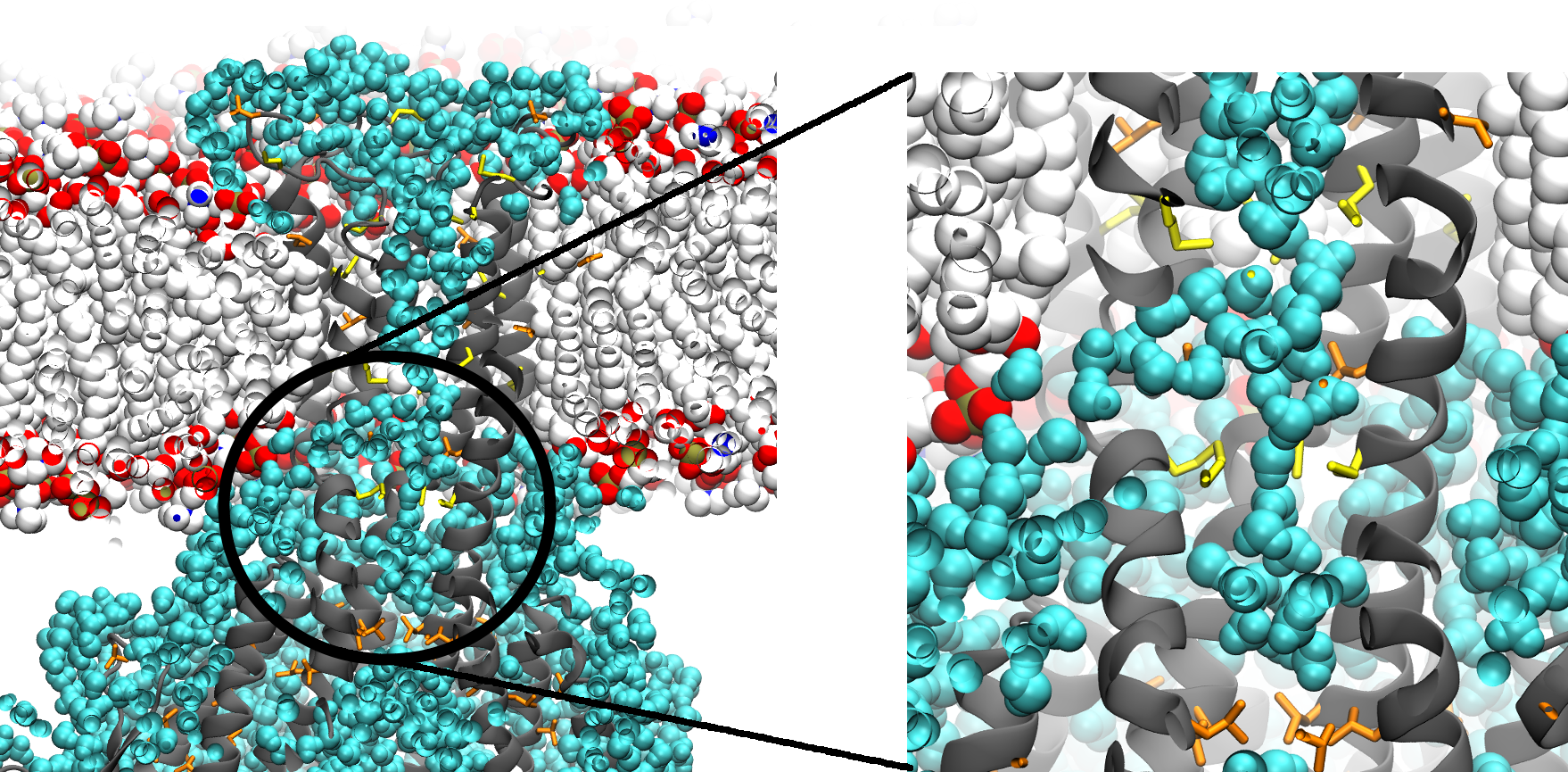

Supplement: S1 Fig — The bilayer is depicted in spheres for (white) carbon, (red) oxygen, (blue) nitrogen, and (brown) phosphorus atoms. Water molecules are shown in cyan. TmCorA is depicted as grey ribbons. Methionine and leucine side chains are shown as yellow and orange sticks, respectively. (TIF) [file pcbi.1004303.s005.tif]

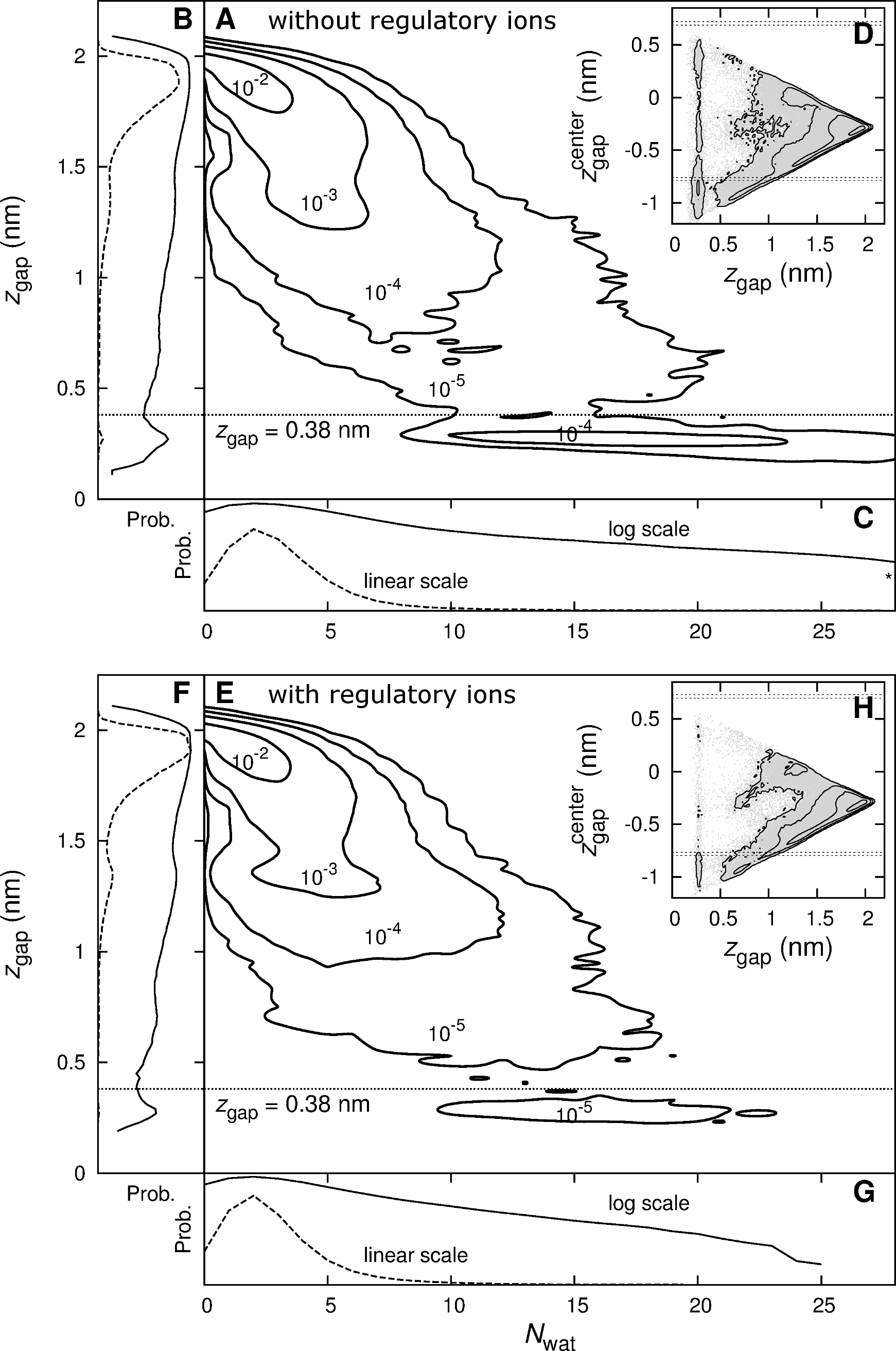

Supplement: S2 Fig — (A and E) Contour plots correlating N wat and z gap (Pearson correlation coefficient of -0.8). (B and F) The probability profile of z gap, in which local minimum at z gap = 0.38 nm is indicated by a horizontal line. (C and G) The probability profile of N wat. In parts B,C,F, and G, both (dashed line) linear- and (solid line) log-scale probabilities are shown, offset for clarity. (*) In part C, probabilities of N wat remain non-zero beyond the edge of the plot. (D and H) Inset contour maps of the location of the center of the local dehydration along z with respect to the center of mass of the MM, zgapcenter, as a function of the length of the dehydrated region. Dotted lines enclose one standard deviation of the positions of the Cα atoms of M291 and M302, the residues which bound the MM. (TIF) [file pcbi.1004303.s006.tif]

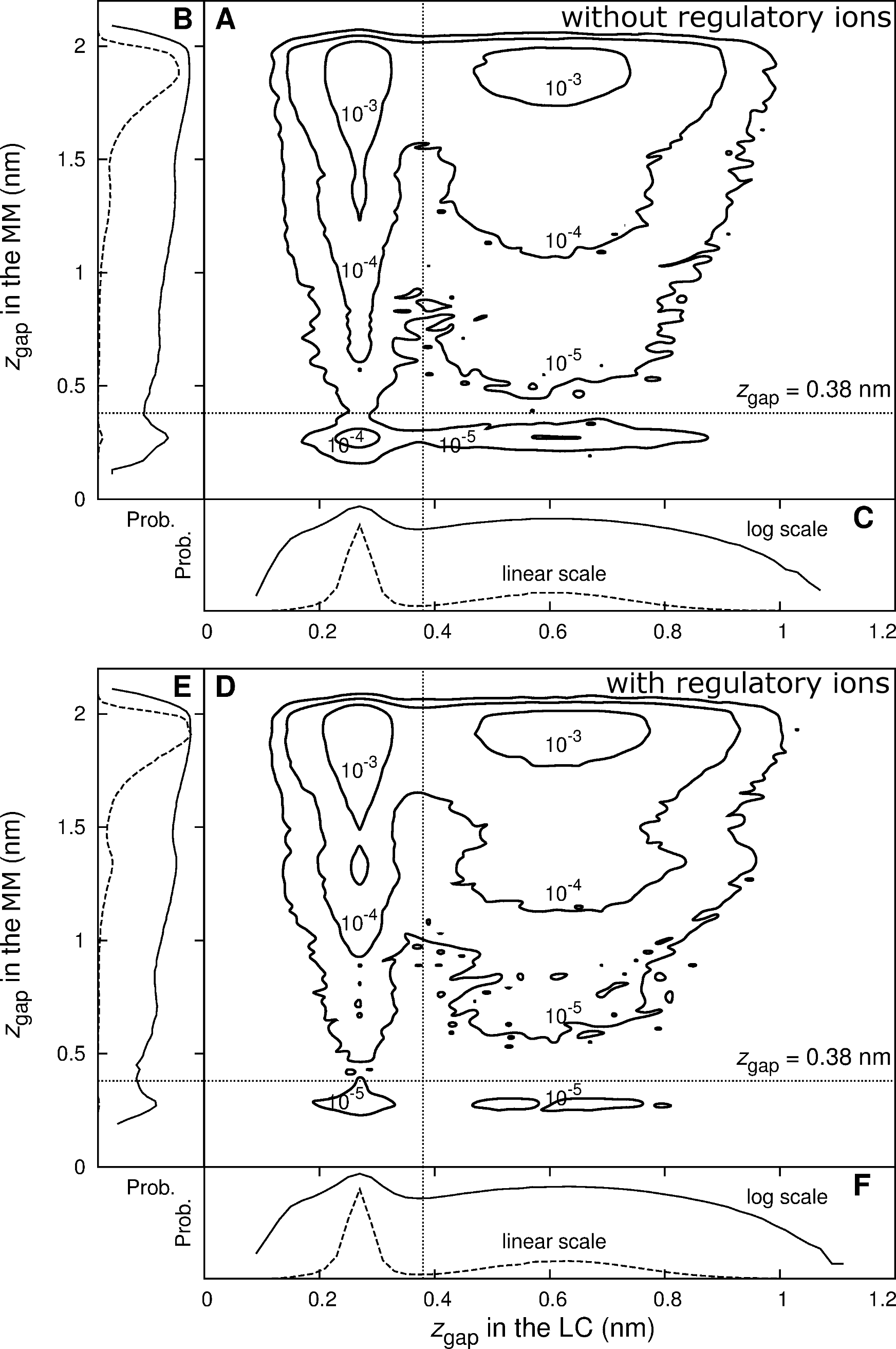

Supplement: S3 Fig — (A and D) Contour plots correlating z gap values in the MM and LC. (B and E) The probability profile of z gap in the MM. (C and F) The probability profile of z gap in the LC. Both (dashed line) linear- and (solid line) log-scale probabilities are shown in parts B,C,E, and F, offset for clarity. A local minimum at z gap = 0.38 nm is indicated by horizontal and vertical dotted lines. (TIF) [file pcbi.1004303.s007.tif]

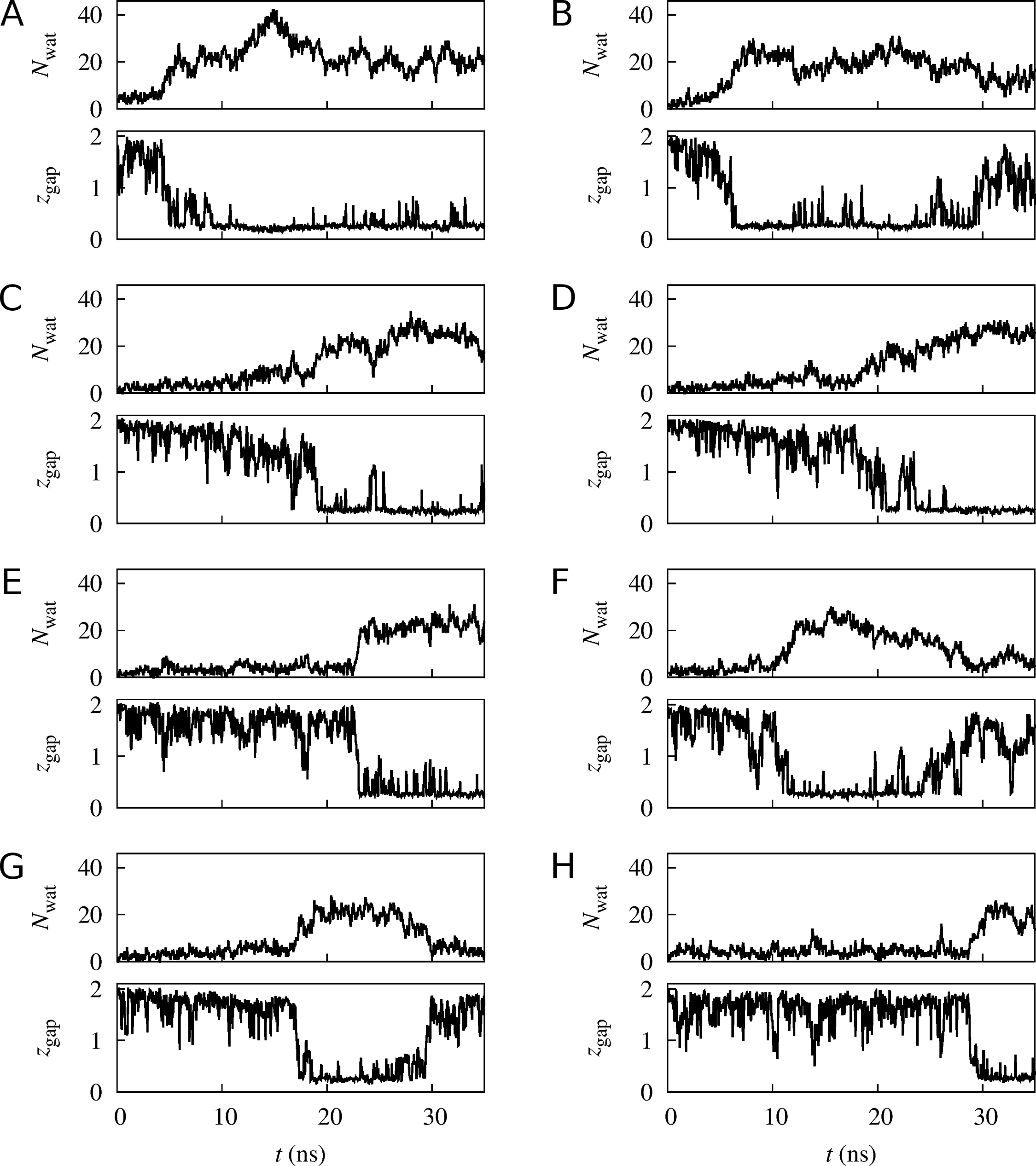

Supplement: S4 Fig — The number of water molecules in the MM, N wat, and the length of the largest dehydration, z gap, are shown. The eight SSH trajectories are ordered A to H by decreasing percent of time spent with N wat > 20. (TIF) [file pcbi.1004303.s008.tif]

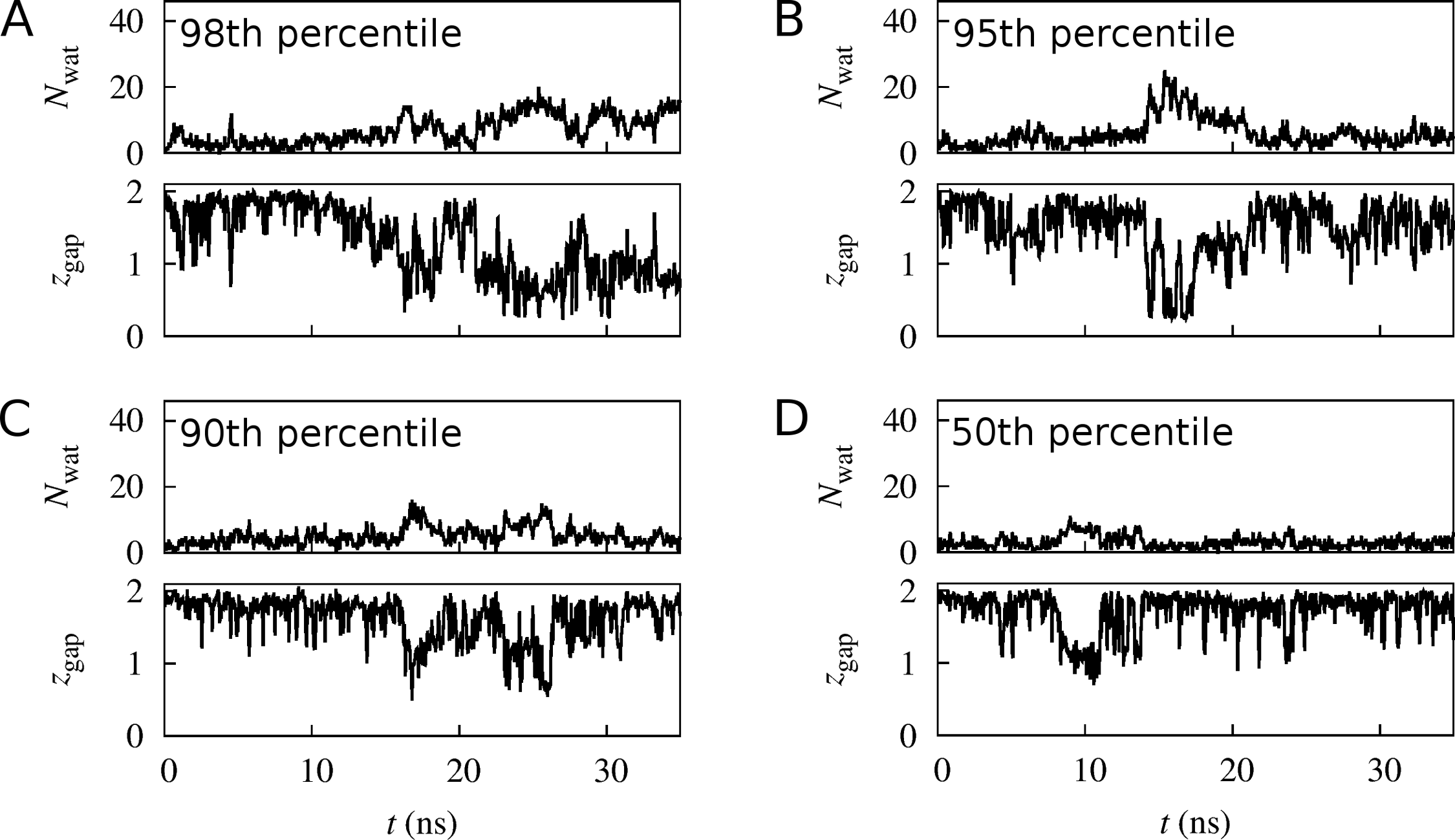

Supplement: S5 Fig — The number of water molecules in the MM, N wat, and the length of the largest dehydration stretch, z gap (nm) are shown. Simulations are ordered by decreasing time spent with N wat > 10 and trajectories are shown from the (A) 98th, (B) 95th, (C) 90th, and (D) 50th percentile. (TIF) [file pcbi.1004303.s009.tif]

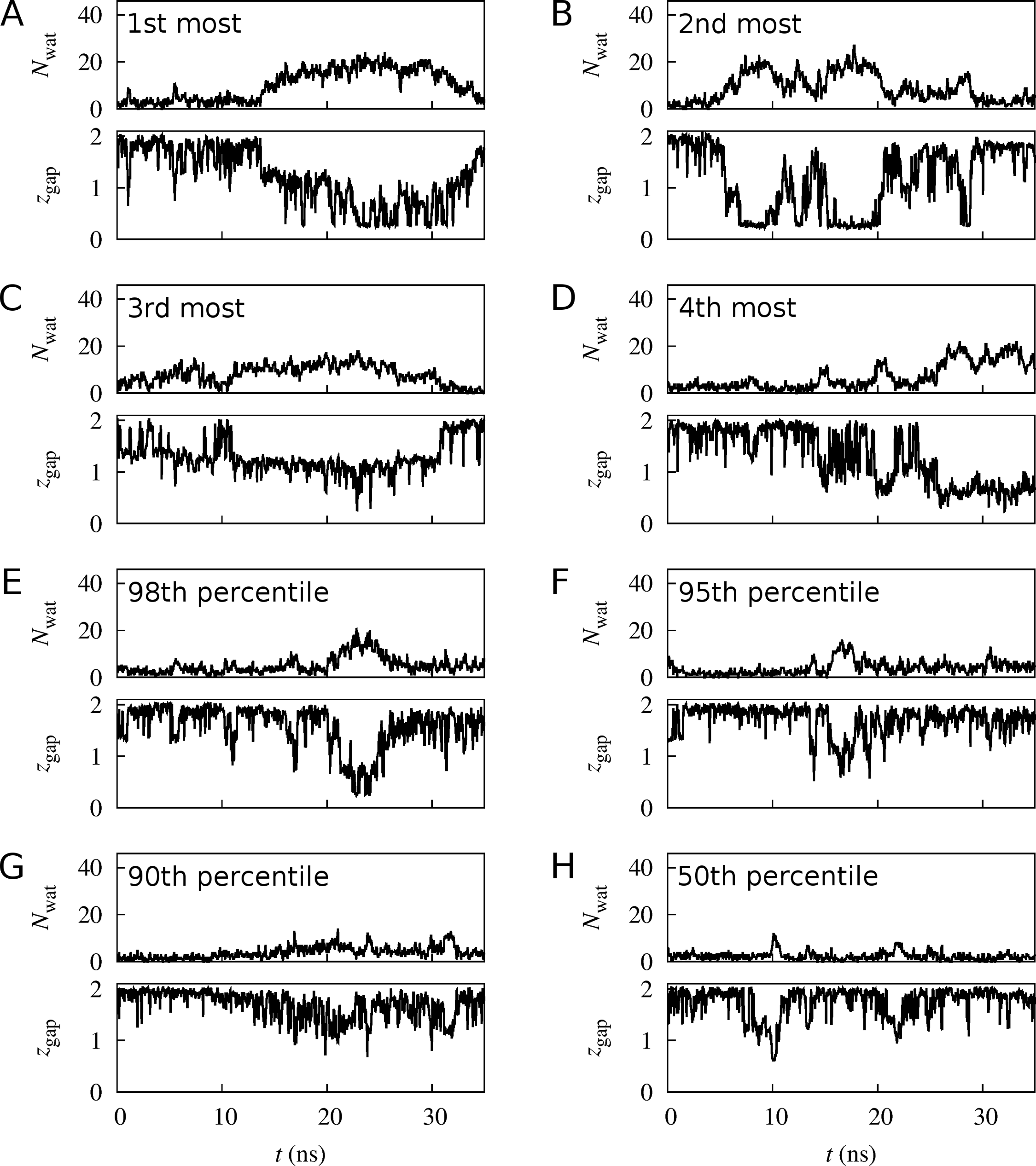

Supplement: S6 Fig — The number of water molecules in the MM, N wat, and the length of the largest dehydration stretch, z gap (nm) are shown for (A-D) the four simulations with the largest time spent with N wat > 10 and (E-H) representative trajectories at the 98th, 95th, 90th, and 50th percentile. (TIF) [file pcbi.1004303.s010.tif]

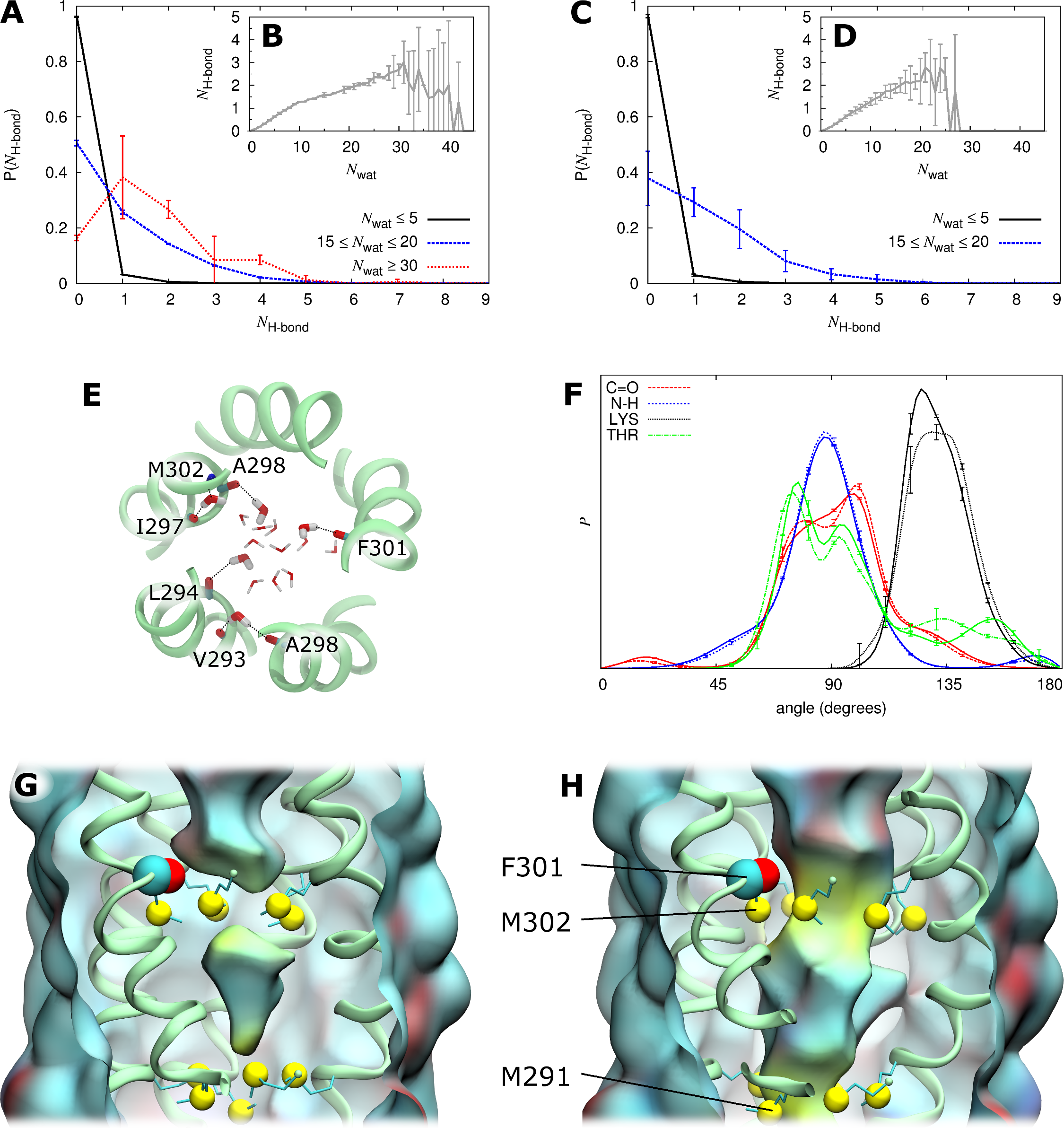

Supplement: S7 Fig — (A-D) Dependence of the number of hydrogen bonds between MM residues and lumenal water molecules, N H-bond, upon the number of water molecules in the MM, N wat, successively (A, B) without and (C, D) with regulatory magnesium ions. (B, D) Insets show the average value of N H-bond as a function of N wat. (E) Extracellular view of TmCorA showing the snapshot with the largest observed value of N H-bond = 7 (not a SSH simulation). Water molecules in the MM that do not engage in water-protein hydrogen bonding are depicted with thinner lines. (F) Orientation of polar backbone groups and side chains of the MM relative to the pore: probability distributions of the (red) backbone O-C-CCOM angle; (blue) backbone H-N-NCOM angle; (black) lysine Nζ-Cα-CαCOM angle; and (green) threonine Oγ-Cα-CαCOM angle (in this notation, the subscript COM indicates the center of mass of the five protomeric atoms at a given location). Solid and patterned lines denote simulations without and with regulatory ions, respectively. The appropriate vectors are computed for all MM residues (M291-M302) on all five protomers and combined into a single histogram. (G, H) View of the MM and its hydration from simulations without regulatory magnesium ions in which the carbonyl oxygen atom of F301 accesses the pore, showing snapshots from the (G) least and (H) most hydrated such simulations. MM methionine Sδ atoms are shown as yellow spheres and the F301 C = O atoms are shown as cyan and red spheres, respectively. The protein's MM is shown as a cartoon with a colored surface. (TIF) [file pcbi.1004303.s011.tif]

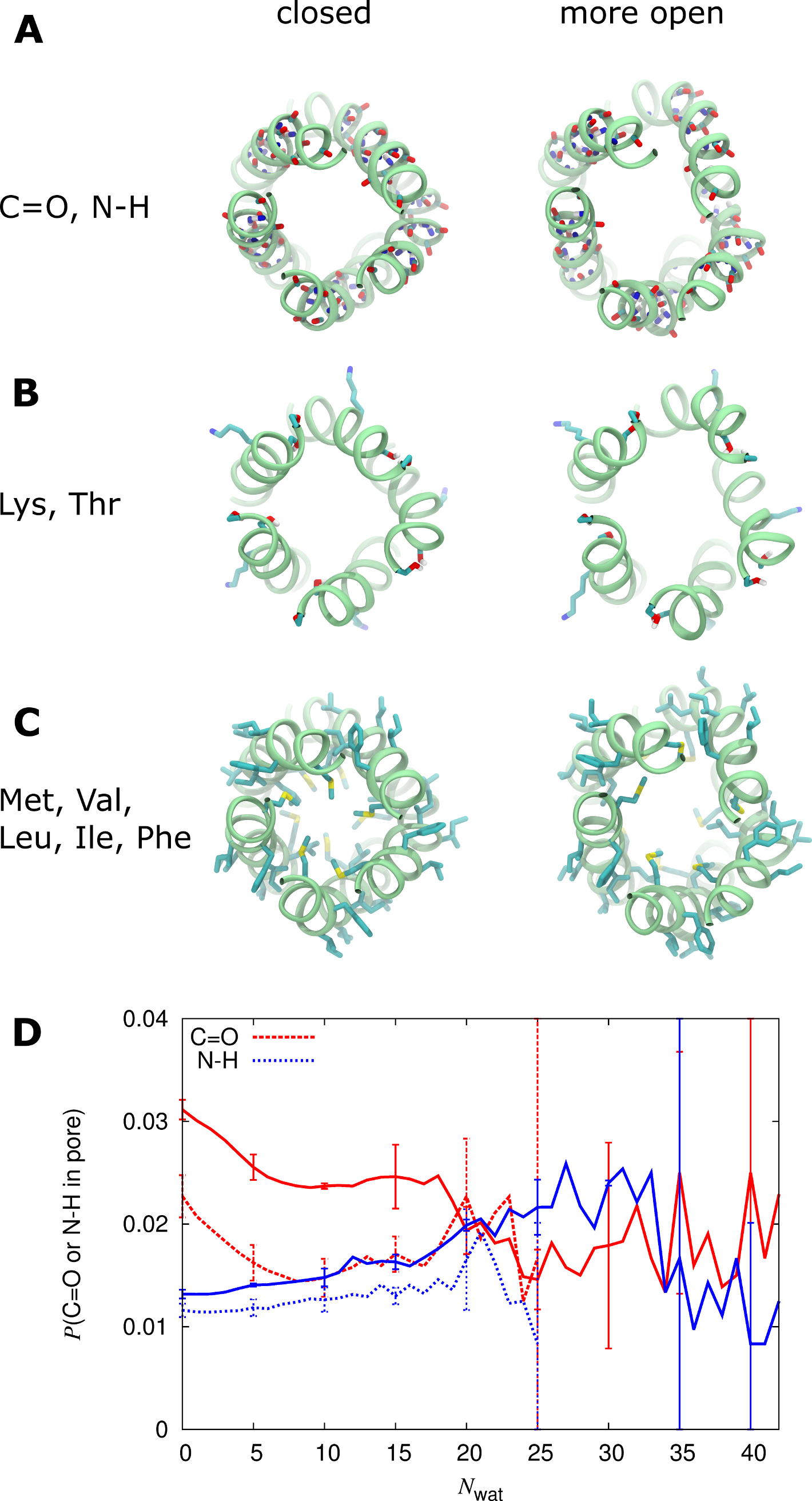

Supplement: S8 Fig — (A-C) Snapshots depict the (left) least and (right) most hydrated simulation without regulatory ions depicting (A) hydrophilic backbone groups, (B) hydrophilic side chains, and (C) hydrophobic side chains along the MM. (D) Probability that the backbone (red) CO or (blue) NH bond vectors project into the pore (angle ≤ 40° as defined in the caption of S7 Fig) as a function of N wat. (TIF) [file pcbi.1004303.s012.tif]

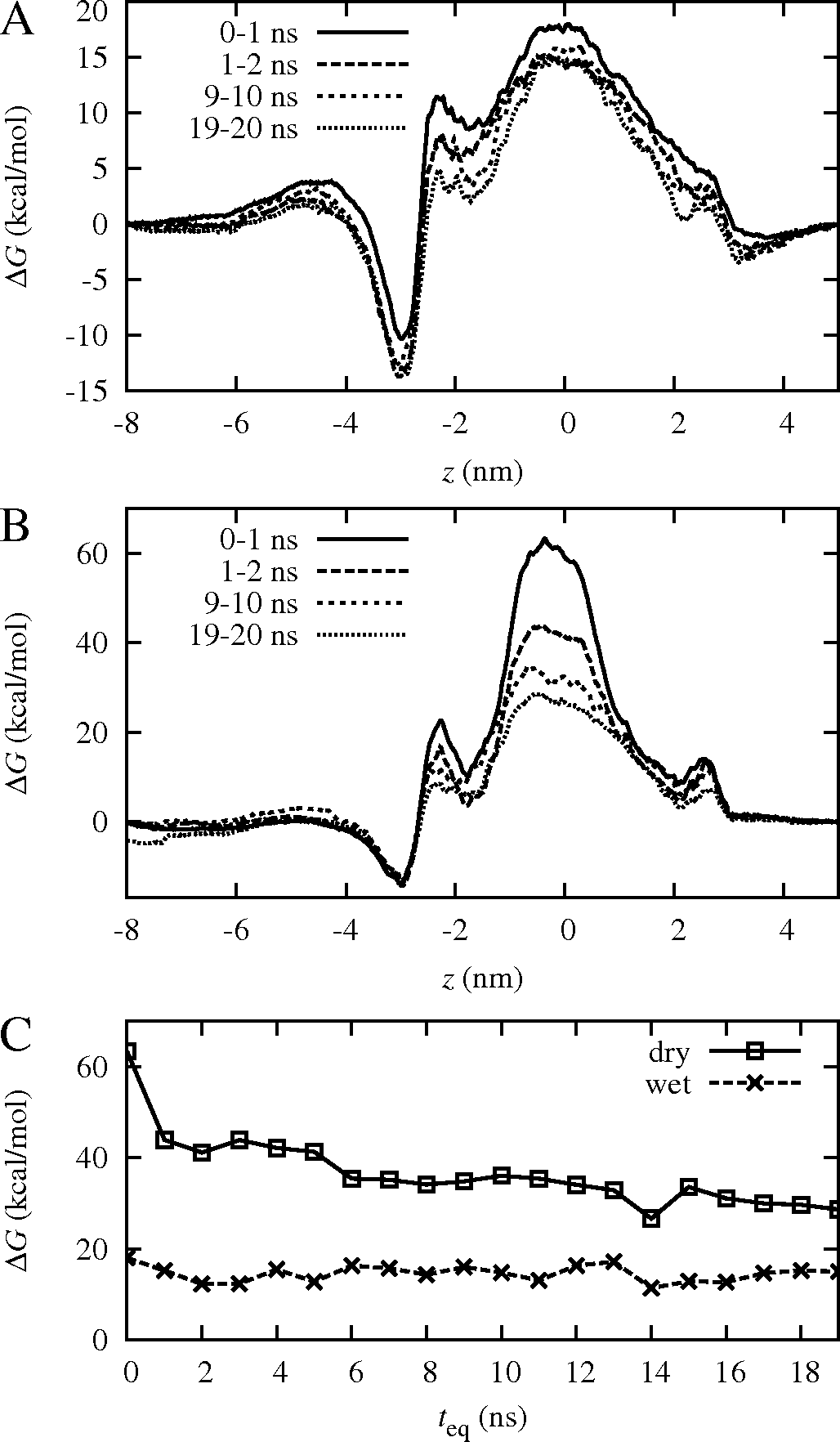

Supplement: S9 Fig — Sampling was initiated using a structure in which the MM was initially (A) wet or (B) dry. Neither of these systems contained regulatory ions. In each case, four PMFs are shown, each computed from 1 ns of sampling per umbrella after an increasing amount of equilibration time per umbrella, t eq, which was either (solid line) 0, (long-dashed line) 1, (short-dashed line) 9, or (dotted line) 19 ns. (C) The magnitude of the free energy barrier in the MM is shown as a function of t eq for the systems in which the MM was initially (“x” symbols and broken line) wet or (open squares and solid line) dry. (TIF) [file pcbi.1004303.s013.tif]

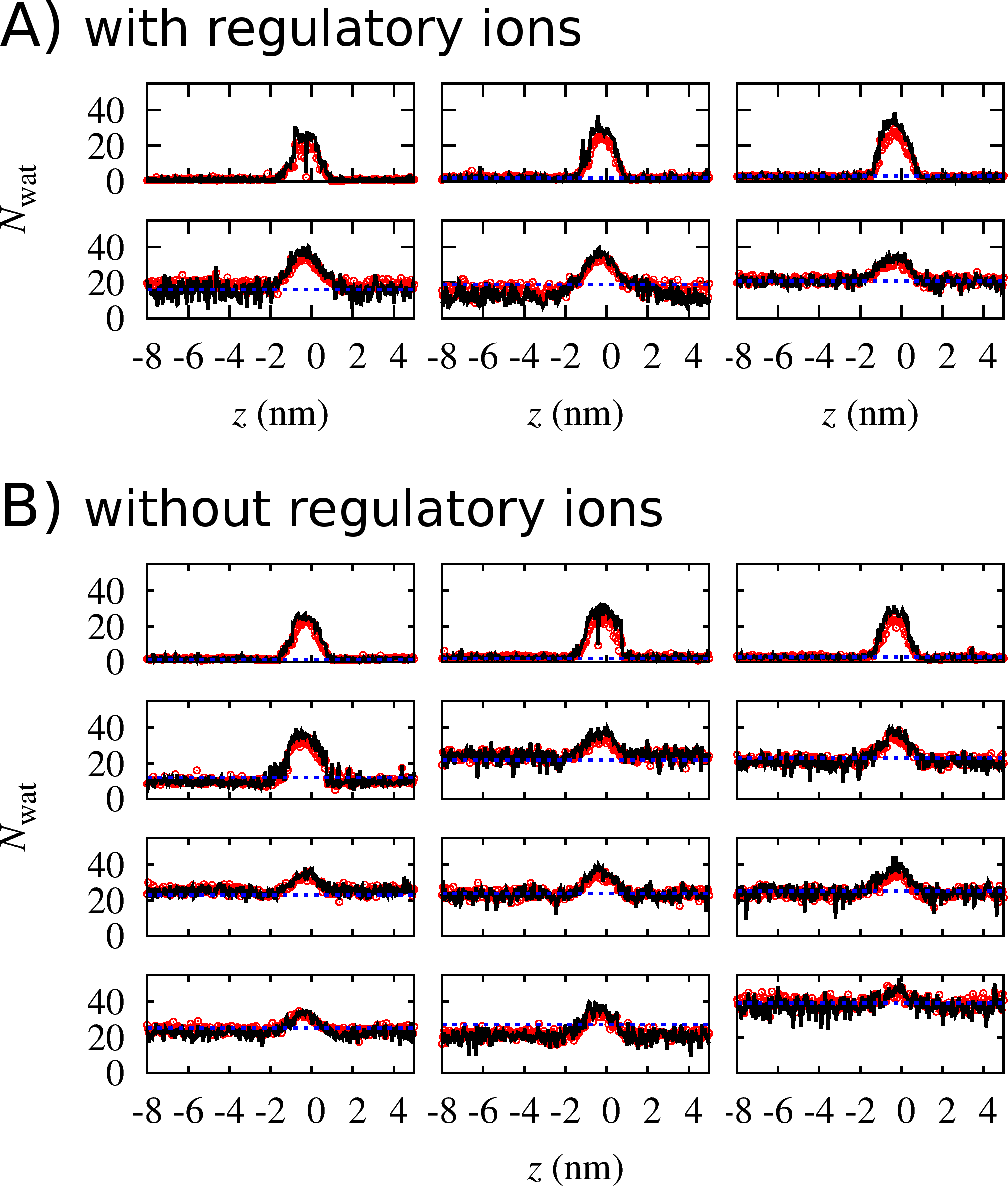

Supplement: S10 Fig — Each plot depicts a set of US simulations initiated with a different starting structure. Dashed blue horizontal lines indicate the number of water molecules initially in the MM. The average values of N wat sampled during US simulations are shown as functions of the position of the lumenal Mg2+ ion along the pore axis, z, which is centered at the center of mass of MM backbone atoms. Values of N wat are averaged over the (red circles) first or (black lines) second ns of simulation/umbrella. Note that the ordinate, N wat, describes hydration of the MM and not the local environment around the lumenal Mg2+ ion. (TIF) [file pcbi.1004303.s014.tif]

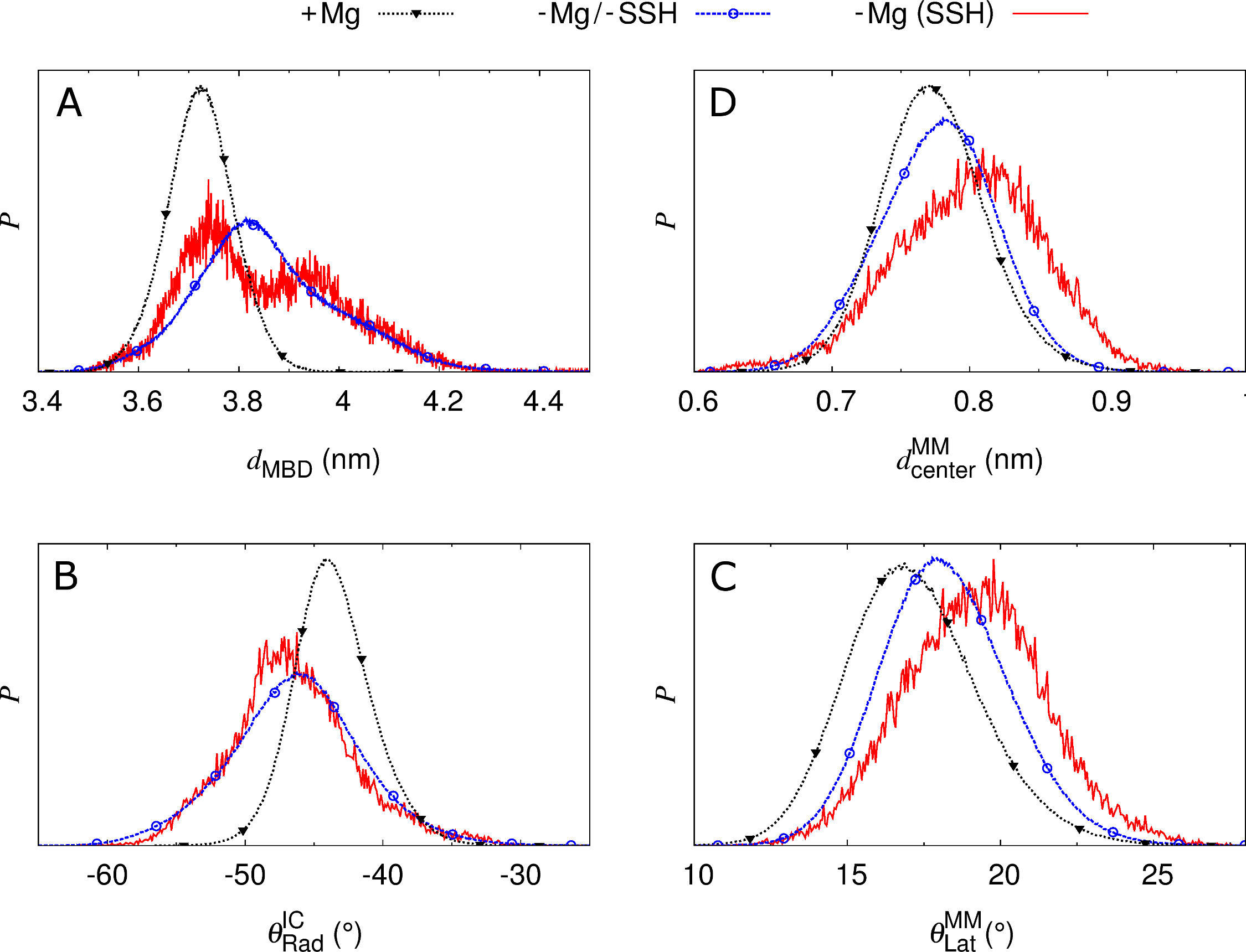

Supplement: S11 Fig — Data are shown for (dotted black line with filled triangles) all 700 simulations with regulatory ions, (dashed blue line with open circles) the 692 non-SSH simulations without regulatory ions, and (red solid line) the 8 SSH simulations identified in Fig 3 for the system with no regulatory ions. Probability distributions of (A) distances between the centers of mass of adjacent cytoplasmic domains, dMBD, (B) radial tilts of the cytoplasmic part of the pore-lining helices, θRadIC, (C) lateral tilts of α7 helices on the pore surface in the MM, θLatMM, and (D) distances of the axes of α7 helices from the center of the pore in the MM, dcenterMM. (TIF) [file pcbi.1004303.s015.tif]

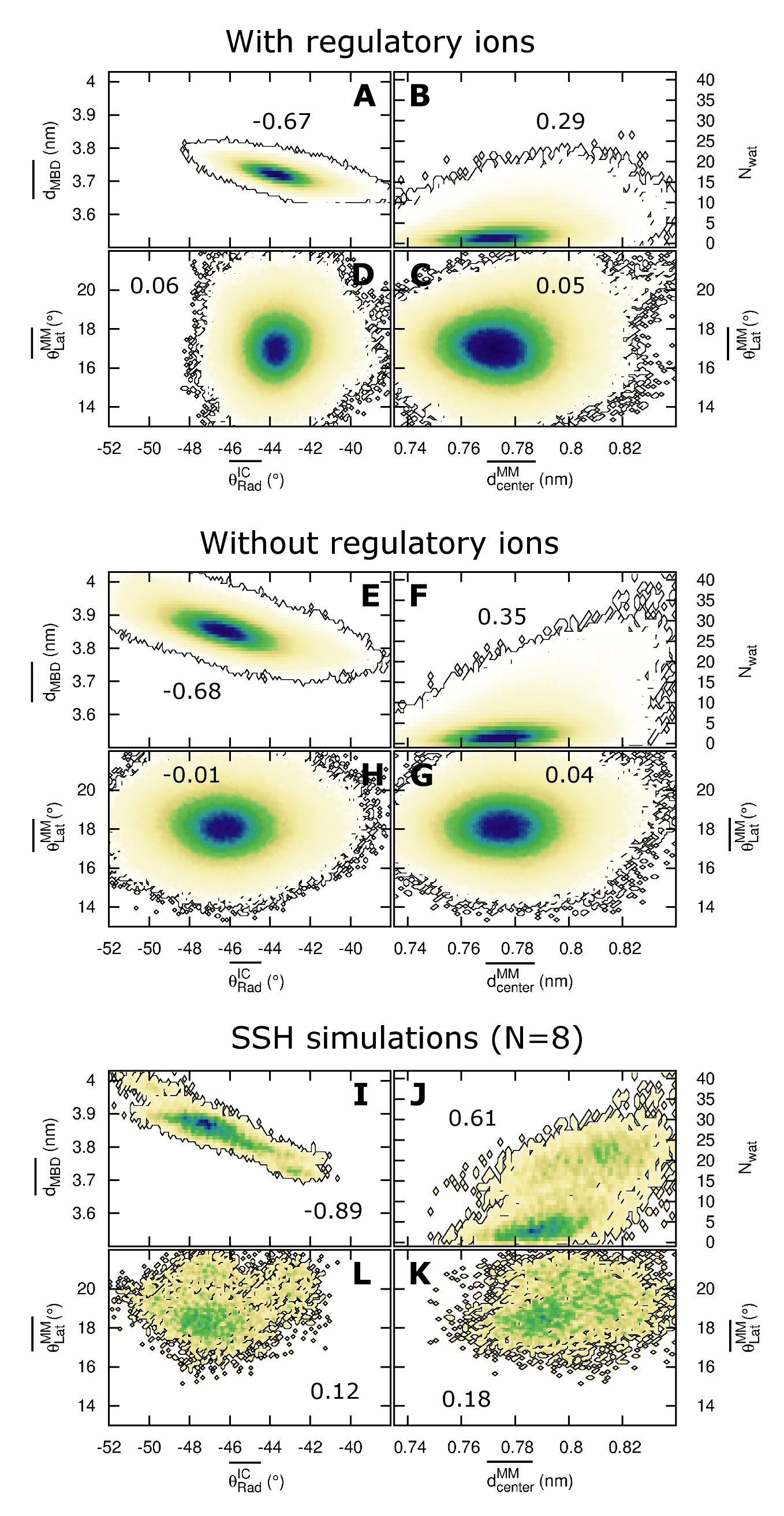

Supplement: S12 Fig — Data are shown for (A-D) all 700 simulations with regulatory ions, (E-H) all 700 simulations without regulatory ions, and (I-L) the 8 stably superhydrated (SSH) simulations identified in Fig 3 for the system with no regulatory ions. Symbols are the same as those in S11 Fig, except that here bars over symbols denote average values from the five protomers. Pairwise correlations are shown for (A,E,I) θRadIC¯ vs dMBD¯, (B,F,J) dcenterMM¯ vs N wat, (C,G,K) θRadIC¯ vs θLatMM¯, and (D,H,L) dcenterMM¯ vs θLatMM¯. Regions of dense sampling are colored blue and regions of sparse sampling are colored yellow. (TIF) [file pcbi.1004303.s016.tif]

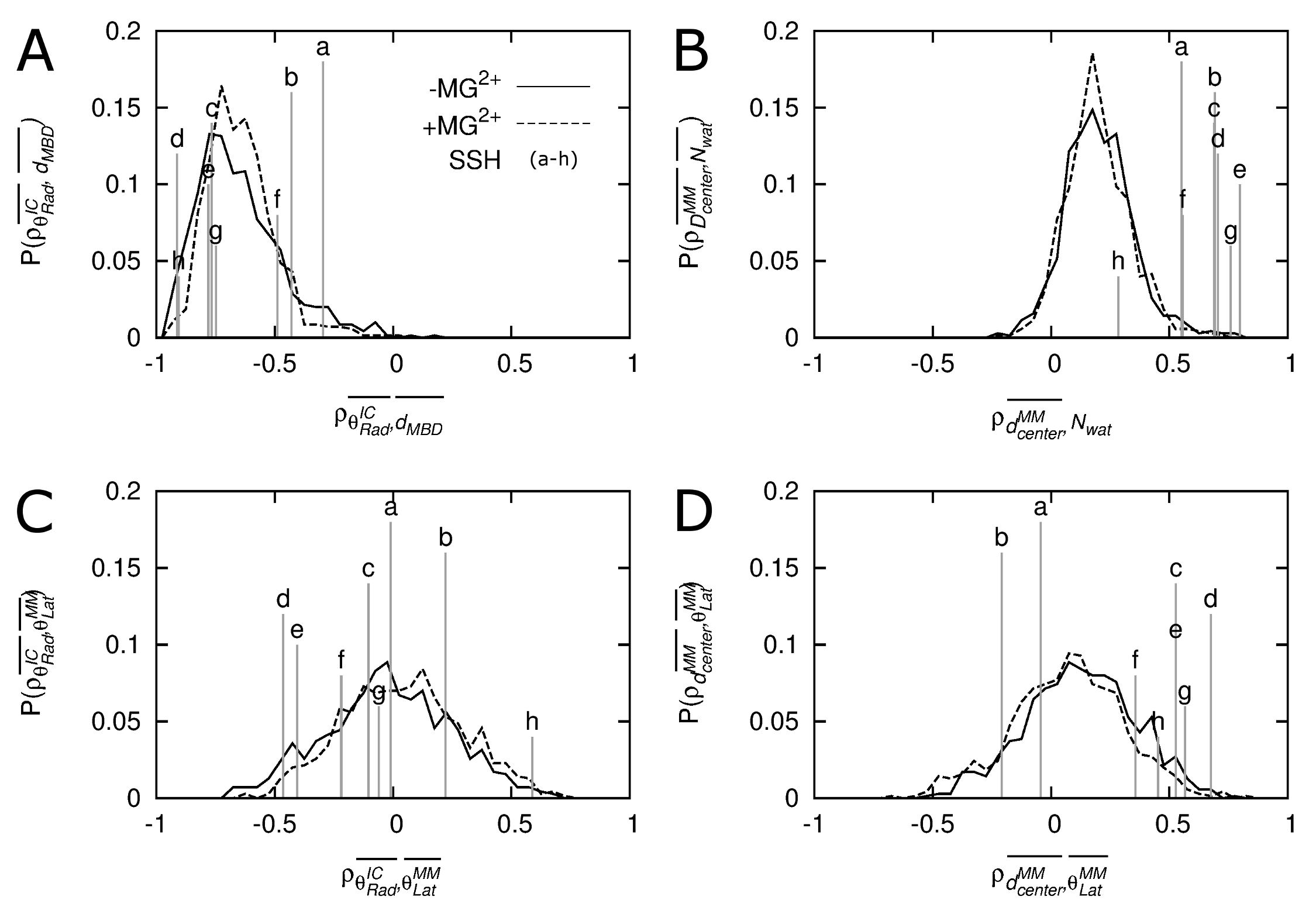

Supplement: S13 Fig — Pearson coefficients, ρ, were computed from individual simulations for the four pairs of metrics considered in S12 Fig. Distributions are shown for (solid line) all 700 simulations without regulatory ions and (broken line) all 700 simulations with regulatory ions. The Pearson correlation coefficient of each of the 8 stably superhydrated simulations is identified in each plot by vertical lines and the letters a-h, which correspond to simulation identifiers in S4 Fig. SSH identifiers are distributed along the ordinate to ease reading, but only contain information in relation to the abscissa. (TIF) [file pcbi.1004303.s017.tif]

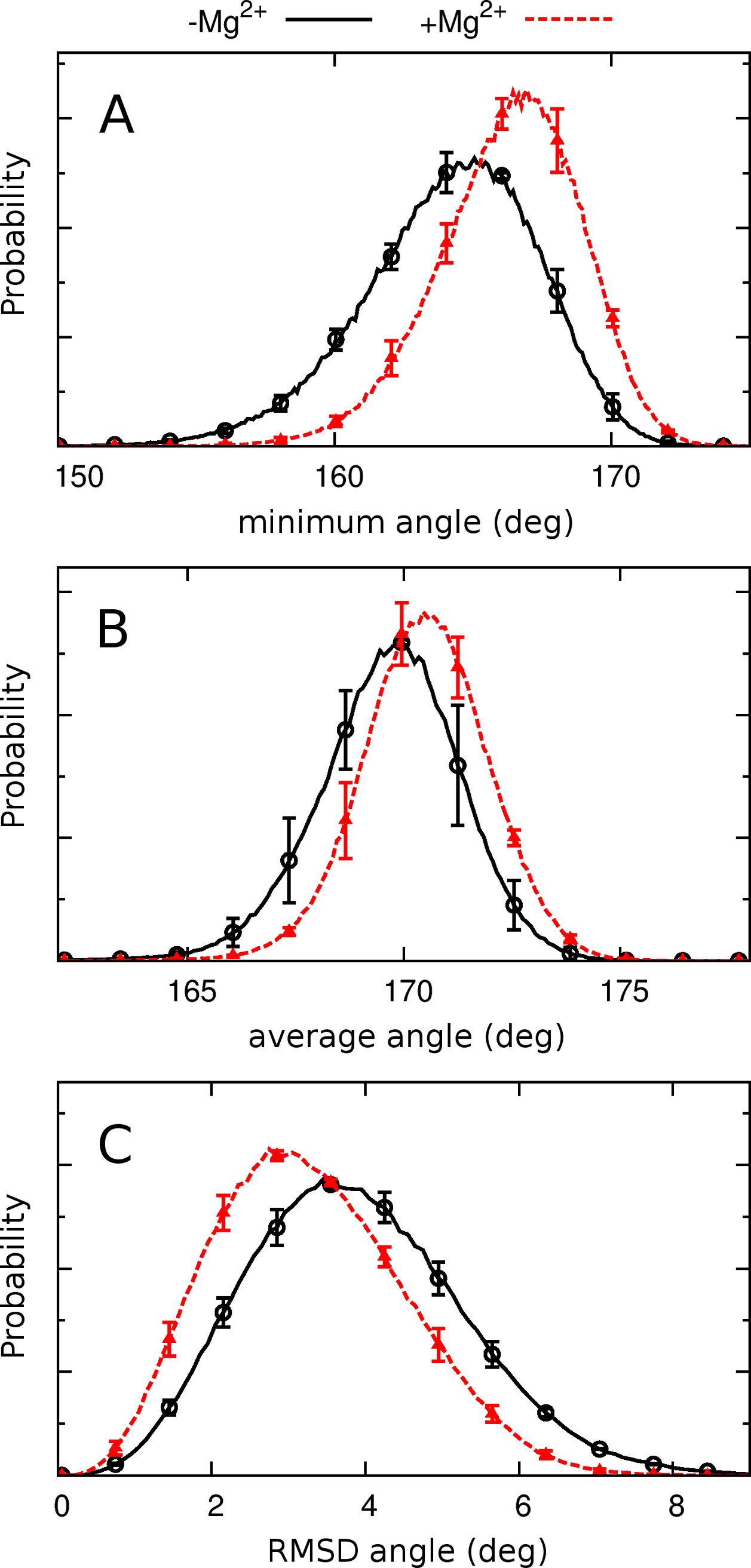

Supplement: S14 Fig — Probability histograms of the instantaneous (A) minimum, (B) average, and (C) root mean squared deviation (RMSD) of the bending angle between V248, L280, and I310 (all Cα) in each protomer for simulations conducted (dashed red lines) with and (solid black lines) without regulatory ions. Standard deviations were obtained by dividing each set of 700 simulations into 2 subsets. (TIF) [file pcbi.1004303.s018.tif]

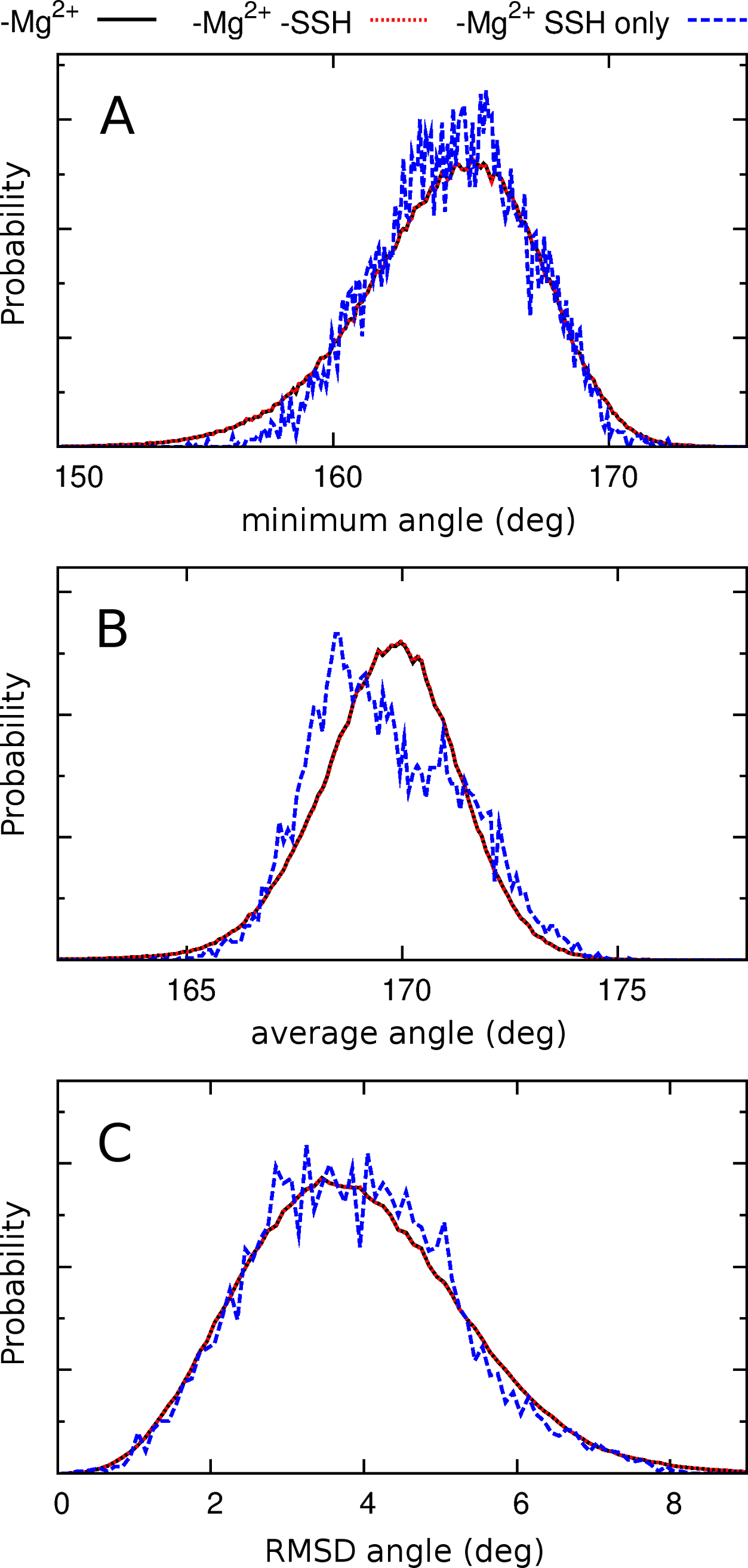

Supplement: S15 Fig — Probability histograms of the instantaneous (A) minimum, (B) average, and (C) root mean squared deviation (RMSD) of the bending angle between V248, L280, and I310 (all Cα) in each protomer for (solid black lines) all simulations, (dotted red line) not including the SSH simulations, and (dashed blue line) only the SSH simulations. The dotted red line appears almost exactly on top of the solid black line. (TIF) [file pcbi.1004303.s019.tif]

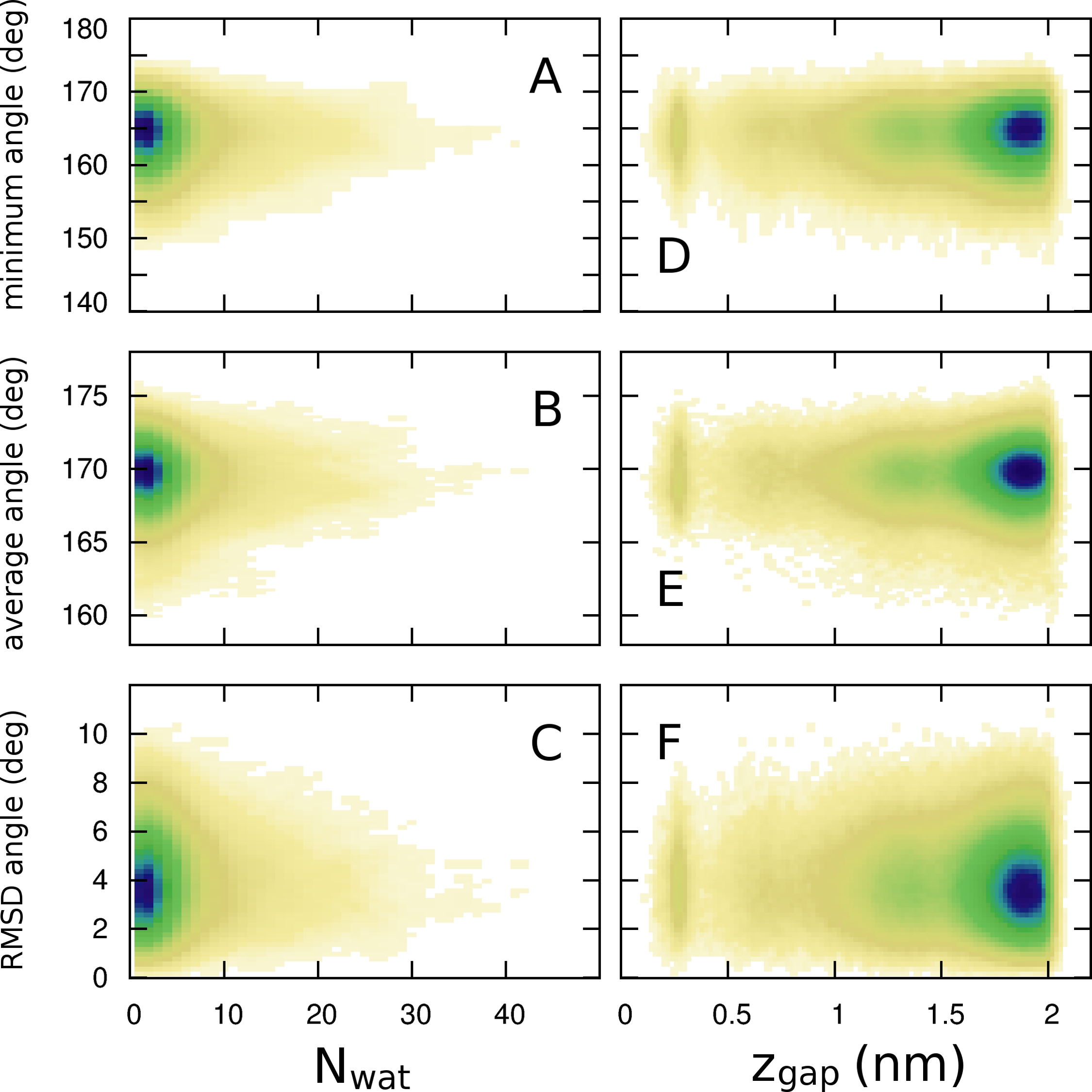

Supplement: S16 Fig — Heat maps evaluate pore hydration via (A-C) N wat and (D-F) z gap and the instantaneous (A, D) minimum, (B, E) average, and (C, F) root mean squared deviation (RMSD) of the bending angle between V248, L280, and I310 (all Cα) in each protomer. (TIF) [file pcbi.1004303.s020.tif]
